# Absence of a prolonged macrophage and B cell response inhibits heart regeneration in *the Mexican cavefish*

**DOI:** 10.1038/s41467-026-77725-4

**Published:** 2026-09-17

**Authors:** Esra Sengul, Helen G. Potts, Ryan D. Carter, William T. Stockdale, Laura Bevan, Maria Nozdrina, Jun Ying, Konstantinos Lekkos, Rita Alonaizan, Zhilian Hu, Abigail Goodship, Lucy O’Byrne, Madeleine E. Lemieux, Rebecca Richardson, Mathilda T. M. Mommersteeg

**Affiliations:** 1 Institute of Developmental and Regenerative Medicine, Old Road Campus, University of Oxford, Oxford, UK; 2 Department of Physiology, Anatomy & Genetics, University of Oxford, Oxford, UK; 3 School of Biochemistry and Biomedical Sciences, University of Bristol, Bristol, UK; 4 King Faisal Specialist Hospital & Research Centre, Riyadh, Saudi Arabia; 5 Bioinfo, Plantagenet, ON Canada

**Keywords:** Self-renewal, Evolutionary developmental biology, Regeneration, Cell death and immune response

## Abstract

A balanced immune response after cardiac injury is crucial to successful heart regeneration, but knowledge of what distinguishes a regenerative from a scarring response is still limited. The Mexican cavefish *Astyanax mexicanus* provides a unique comparative model to study heart regeneration and scarring within a single species. *Astyanax* surface-dwelling fish are capable of heart regeneration, whereas, like the human heart, their cave-dwelling Pachón counterparts form a permanent scar. Using single-cell transcriptomics and immune perturbations, we identify two distinct and largely sequential immune mechanisms driving regeneration in surface fish. First, late-present macrophages are actively phagocytic, and pharmacological inhibition of phagocytosis disrupts scar clearance and regeneration. Second, B cells recruited from 7 days post-injury promote cardiomyocyte cell progression into mitosis, and their inhibition with Acalabrutinib significantly impairs cardiomyocyte proliferation and regeneration. This B cell-dependent mechanism is conserved in zebrafish. Our findings reveal two independent late immune mechanisms distinguishing regenerative from non-regenerative cardiac repair, offering insights for potential therapeutic strategies to enhance heart regeneration in mammals.

## Abstract (web summary)

The immune response is important after cardiac injury and may mediate scarring vs. regeneration. Here they show that the regenerative response involves a two-phase immune response in regenerative Mexican cavefish that is not seen in non-regenerative cavefish but partially shared with zebrafish.

## Introduction

Regeneration of the adult heart is a remarkable ability seen in some species, such as zebrafish, newts and axolotls^1–4^. This response is absent in humans, where injury to the heart often results in scarring and permanent damage. After myocardial infarction (MI), the human heart undergoes an acute inflammatory response as dying cells release damage-associated molecular patterns, triggering immune cells to release cytokines and chemokines^5^. This cascade activates a host of immune responses aimed at repairing the damaged tissue, but also causing excessive inflammation, extracellular matrix (ECM) remodelling and scar formation rather than regeneration^6,7^. Over time, this fibrotic response can compromise cardiac function and lead to heart failure. Yet, the immune response has also been shown to be essential for successful regeneration in species such as zebrafish and the neonatal mouse^8,9^.

In both regenerative and non-regenerative species, innate immune cells such as neutrophils and macrophages infiltrate the injury site, initiating an acute inflammatory response. However, in regenerative species, these immune components play critical roles in clearing debris, promoting angiogenesis and stimulating cardiomyocyte proliferation, one of the hallmarks of successful regeneration^10–12^. It is striking how the same immune components can either hinder or support successful regeneration, relying on a delicate, tightly regulated balance. For instance, prolonged neutrophil activity can impair healing, while macrophages can promote either regeneration or drive scarring, depending on their activation state and timing^9,13,14^.

Adaptive immune cells have also been implicated in heart regeneration, with emerging evidence suggesting that B cells may influence cardiomyocyte proliferation and modulate inflammation, making them an intriguing focus for regenerative studies^15–18^. However, their role in the regenerative process has not yet been fully elucidated, and further research is needed to determine whether targeting B cell activity could enhance cardiac repair. Understanding the immune response that enables successful regeneration in certain species versus scarring in others may provide valuable insights for designing therapeutics to enhance post-MI cardiac repair in humans.

To explore the interplay between immunity and heart regeneration, the Mexican cavefish, *Astyanax mexicanus*, offers a compelling model. This species exists in two main morphs: surface-dwelling fish, capable of regenerating their hearts after injury and cave-dwelling fish, of which at least one cavefish population has lost this regenerative capability and instead forms a permanent scar^19^. The cave-dwelling fish have evolved in the isolated, nutrient-deficient and low-diversity environments of Mexico’s underground caves^20,21^. They are blind, with reduced immune activity due to the absence of light and changes in pathogen exposure^22^. This unique evolutionary background provides a natural framework for studying the genetic and immune differences that influence natural regenerative outcomes without the complications of cross-species comparisons^23^.

In this study, we use a multidimensional approach to dissect the role of immune mechanisms during cardiac regeneration, comparing the surface fish against their cave-dwelling Pachón counterparts. Our findings reveal that surface fish exhibit a prolonged, balanced immune response, characterised by a sustained presence of both myeloid and lymphoid populations, that supports regeneration, while Pachόn fish show a rapid but transient response that culminates in scarring. Inhibition of this prolonged inflammation in surface fish with a broad-acting anti-inflammatory drug, Dexamethasone, results in reduced cardiomyocyte proliferation and impaired regeneration. A detailed characterisation of the immune cell populations between surface fish and Pachón fish shows that the difference in leukocyte numbers is driven by higher numbers of macrophages and B cells in the surface fish during the later recovery stages. Late-stage phagocytic macrophages are required for scar clearance, as demonstrated by both Clodronate liposome depletion and pharmacological inhibition of phagocytosis using 2-acetamidophenol. Independently, B cells in surface fish increase in number from 7-days post cryoinjury (dpci) and promote cell cycle progression into mitosis, whilst their inhibition with BTK inhibitor Acalabrutinib significantly impairs cardiomyocyte proliferation and regeneration. The *rag* zebrafish mutant supports these findings, as the lack of mature lymphocytes impairs regeneration by hindering cardiomyocyte proliferation.

Collectively, these findings reveal two independent immune mechanisms that distinguish regenerative from non-regenerative cardiac repair, pointing to specific cellular targets for therapeutic intervention in regenerative medicine.

## Results

### Inhibition of prolonged inflammation impairs heart regeneration

**Fig. 1:**
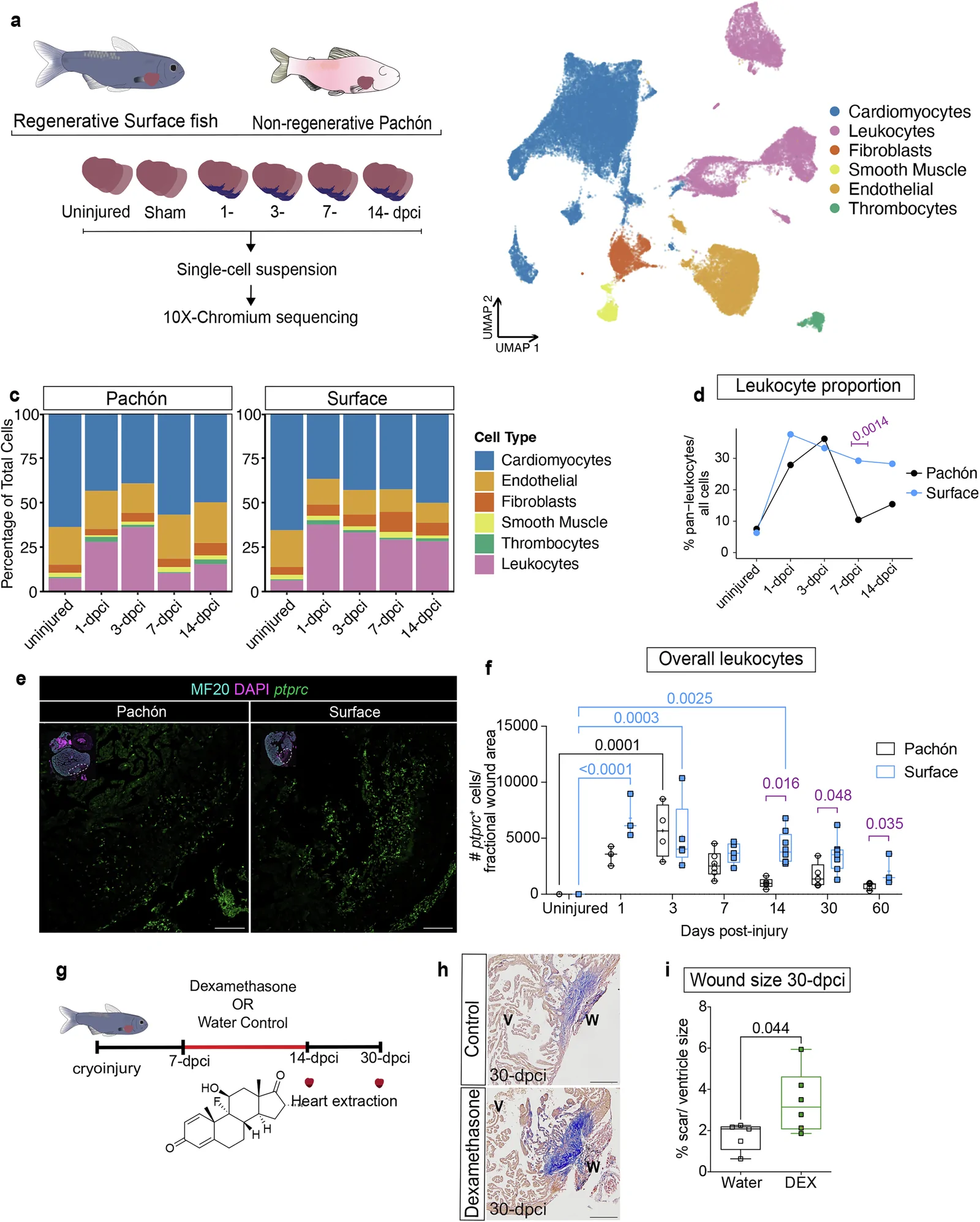
Prolonged inflammation after cardiac injury is required for successful repair in surface fish hearts. **a** Pachόn and surface fish ventricles were collected uninjured, 3-dps, 1-, 3-, 7- and 14-dpci, and 3 hearts per group per condition were pooled. The tissues dissociated into single cells and processed for 10X-Chromium single-cell RNA sequencing. **b** Annotated UMAP showing all the expected cell types of the heart. **c** Stacked-bar charts showing the proportion of major cardiac cell types as a percentage of total cells for Pachόn and surface fish hearts across all time points. **d** Differential proportion analysis on leukocytes at different time points. Pachόn leukocytes show 4.3× fold change at 1-dpci and 5.60× fold change at 3-dpci above uninjured before returning toward baseline levels. In surface fish, leukocyte fractions elevated across all timepoints (7.73× at 1-dpci, 6.8× at 3-dpci, 5.5× at 7-dpci and 4.59× at 14-dpci). Two-sided permutation analysis at 7-dpci (FDR = 0.0015) and 14-dpci (FDR = 0.0030) with 95% bootstrap confidence intervals above zero at both timepoints. **e** RNAscope targeting *ptprc*^+^ in Pachón and surface fish ventricles at 14-dpci, counterstained with MF20 (cyan) and DAPI (magenta). The dashed line indicates the wound. **f** Quantification of *ptprc*^+^ cells in Pachón and surface at uninjured, 1-, 3-, 7-, 14-, 30- and 60-dpci. *p* values in black indicate comparisons within Pachόn, blue ones within surface fish and purple ones between surface fish and Pachόn. Two-way ANOVA with non-parametric Mann–Whitney-*U* test. Between-group comparisons (Pachón vs Surface) at each timepoint: uninjured (*n* = 3 vs 3, *p* = 1.00), 1 dpci (*n* = 3 vs 3, *p* = 0.10), 3 dpci (*n* = 4 vs 5, *p* = 0.730), 7 dpci (*n* = 6 vs 5, *U* = 7.00, *p* = 0.177, ns), 14 dpci (*n* = 5 vs 8, *U* = 0.00, *p* = 0.0016, *p* < 0.01), 30 dpci (*n* = 5 vs 7, *p* = 0.048), 60 dpci (*n* = 5 vs 3, *p* = 0.036). Total *n*: Pachón = 31, Surface = 34. Boxplots show the median; individual points are overlaid. **g** Experimental schematic: surface fish were exposed to Dexamethasone (10 mg/L) from 7- to 14-dpci. Hearts were collected at 14-dpci and 30-dpci. **h** AFOG-stained sections at 30-dpci. Blue staining indicates collagen-rich scar tissue. **i** Quantification of wound area as a percentage of total ventricle size in control and Dexamethasone-treated surface fish at 30-dpci, two-sided Welsch test, *p* = 0.044, *n* = 6 each group. All scale bars, 100-μm. Source data for **d**, **f**, **i** are provided as a Source Data file. DEX, Dexamethasone; dpci, days post cryo-injury; dps, days post sham; V, ventricle; W, wound.

To characterise the immune response in the regenerative and scarring settings, we performed cryoinjury on surface fish and Pachόn hearts. Ventricles were collected at 1-, 3-, 7- and 14-dpci, as well as uninjured and 3 days-post sham (dps) controls. Samples were processed for 10X Chromium single-cell RNA-sequencing (scRNA-seq), yielding a sequencing depth of ~50,000 reads per cell (Fig. 1a). We identified all expected cardiac cell types as defined by canonical marker genes established in zebrafish and previous studies^24–26^ (Fig. 1b and Supplementary Fig. 1a, b) and examined leukocyte dynamics by sub-setting cells expressing canonical markers *ptprc*, *lcp1* and *cxcr4b* (Supplementary Fig. 1c–e). Plotting the proportion of immune cells relative to other major cardiac cell types revealed that the immune response diverged between regenerative surface fish and scarring Pachόn hearts, particularly at late stages after injury (Fig. 1c). Differential proportion analysis (DPA) showed that Pachόn leukocytes were elevated at 1- and 3-dpci, while surface fish leukocytes were elevated at all time points compared to uninjured levels, including at 7- and 14-dpci (Fig. 1d). We confirmed these scRNA-seq findings on sections using an RNAscope in situ hybridisation probe targeting the pan-leukocyte marker *ptprc* (Fig. 1e, f). After an initially similar response, there was a decline in numbers towards baseline in Pachón wounds after 3-dpci. This is expected after the initial peak as observed in other non-regenerative species^6,27^. However, leukocyte numbers remained high in surface fish, with significantly higher numbers than in Pachón at 14-dpci (Fig. 1f), suggesting that prolonged inflammation is a key factor contributing to cardiac repair.

To test whether this late immune response benefits regeneration, we used the broad-spectrum anti-inflammatory agent Dexamethasone^28^ to suppress the late-stage immune response in surface fish. Fish were treated with Dexamethasone from 7- to 14-dpci (Fig. 1g). Hearts were isolated at 30-dpci, and wound size was analysed using AFOG staining, revealing significantly larger scars in Dexamethasone-treated hearts compared to untreated controls (Fig. 1h, i). These findings highlight the importance of a sustained immune response in promoting effective cardiac repair in surface fish.

### Prolonged innate and adaptive responses in surface fish

**Fig. 2:**
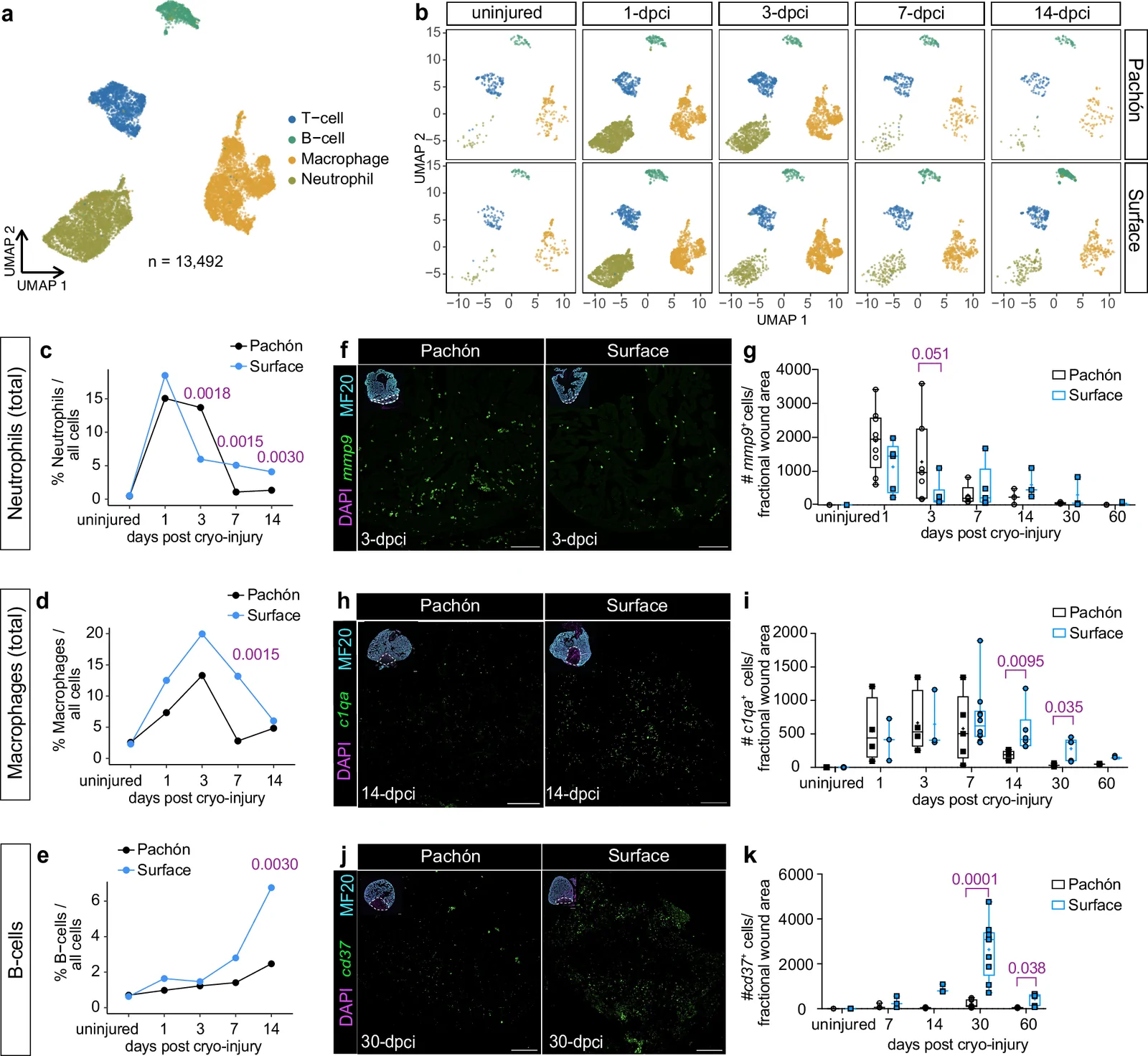
Surface fish have a prolonged innate and adaptive response. **a** Annotated UMAP showing all four main leukocyte populations in Pachόn and surface fish hearts following sub-clustering of leukocytes (*n* = 13,492 cells). **b** UMAPs showing all leukocyte clusters over time in Pachόn (top) and surface fish (bottom) at uninjured, 1-, 3-, 7- and 14-dpci, highlighting that leukocyte numbers in Pachόn decrease at late stages of wound healing, while all clusters remain present. Two-sided permutation-based differential proportion analysis on neutrophils (**c**), monocytes-macrophages (**d**) and B cells (**e**) as a percentage of total cells, at uninjured and at 1-, 3-, 7- and 14-dpci, showing differences between Pachόn and surface fish ventricles. Purple FDR values indicate significant differences between surface fish and Pachόn at the indicated timepoints, two-sided permutation analysis. Representative RNAscope images to show *mmp9*^+^ neutrophils at 3-dpci (**f**), *c1qa*^+^ macrophages at 14-dpci (**h**) and *cd37*^+^ B cells at 30-dpci (**j**), in Pachόn (left) and surface fish (right). All sections were counterstained with MF20 (cyan) and DAPI (magenta). The dashed circle indicates the zoomed-in wound region. Scale bar, 200-μm. Quantification of *mmp9*^+^ (**g**), *c1qa*^+^ (**i**), *cd37*^+^ (**k**) cells normalised to fractional wound area in Pachόn and surface fish across the time course, Pachón *n* = 3 (uninjured, 30- and 60-dpci), *n* = 4 (1-, 3-, 14-dpci), *n* = 5 (7-dpci); Surface *n* = 3 (unijured, 1-, 3-, 60-dpci), *n* = 10 (7-dpci), *n* = 6 (14-dpci), *n* = 5 (30-dpci) **i** and significantly higher B cell numbers from 30-dpci onwards (**k**). No significant differences in neutrophil numbers, Pachón *n* = 3 (uninjured, 14-, 60-dpci), *n* = 9 (1-dpci), *n* = 7 (3-dpci), *n* = 5 (7-dpci), *n* = 4 (30-dpci); Surface *n* = 3 (uninjured, 14-, 30- and 60-dpci), *n* = 5 (1- and 7-dpci), *n* = 6 (3-dpci). **g** *p* = 0.0051 at 3-dpci shown for reference of the marked increase. Purple *p*-values indicate comparisons between surface fish and Pachόn, Pachón *n* = 3 (uninjured, 7-, and 14-dpci), *n* = 8 (30-dpci), *n* = 4 (60-dpci); Surface *n* = 3 (uninjured, 7-, and 14-dpci), *n* = 9 (30-dpci), *n* = 6 (60-dpci). All tests: two-sided Mann–Whitney *U* test. Boxplots show the median and the whiskers extending to the minimum and maximum values; individual points are overlaid. Source data for **g**, **i**, **k** are provided as a Source Data file. dpci: days post cryo-injury.

Since the overall leukocyte response was prolonged in surface fish hearts compared to Pachόn, we further sub-clustered the leukocytes to investigate which immune cell populations were driving this difference (Fig. 2a). We identified the expected leukocyte sub-types with neutrophils, macrophages, B cells and T cells, present in both surface fish and Pachόn across all timepoints (Fig. 2b). These leukocyte populations were validated using a panel of canonical marker genes (Supplementary Fig. 2a–d). Differential proportion analysis highlighted neutrophils, macrophages and B cells as the most distinct between surface fish and Pachόn, while T cells showed no significant differences between morphs across the injury time course and were therefore not investigated further (Fig. 2c–e and Supplementary Fig. 2e).

Neutrophils, among the first immune cells to arrive at the site of injury, peaked at 1-dpci in both fish populations. In contrast to surface fish, numbers remained high in Pachόn until 3-dpci but declined sharply after, whereas in surface fish, neutrophil numbers were more moderately elevated at later stages. The timely resolution of neutrophils is vital for proper wound healing and for preventing chronic inflammation^27,29,30^, and the observed delay in clearance in Pachόn could lead to impaired healing or fibrosis. RNAscope for the neutrophil marker *mmp9* confirmed the strong initial influx of neutrophils into the wound at 1-dpci. However, there were no significant differences in *mmp9*^+^ cell numbers between the two morphs across the time course (Fig. 2f, g). This likely reflects the fact that RNAscope quantification targets the cells within the wound region specifically, whereas scRNA-seq data derives from whole ventricles.

Macrophages peaked similarly at 3-dpci in both Pachόn and surface fish (Fig. 2d). However, surface fish showed significantly higher macrophage levels compared to Pachόn at 7-dpci, suggesting that macrophages are retained at late stages in regenerating hearts. RNAscope analysis for macrophage marker *c1qa* confirmed that surface fish exhibited significantly higher macrophage levels than Pachόn at 14- and 30-dpci (Fig. 2h, i). This suggests that macrophages contribute to the prolonged immune response, potentially supporting debris clearance and cytokine production necessary for tissue repair.

Finally, B cells displayed a strikingly different pattern to neutrophils and macrophages. Both surface fish and Pachόn had minimal B cell numbers at early stages, but surface fish showed a robust increase after 7-dpci, increasing towards 14-dpci (Fig. 2e). RNAscope with pan-B cell marker *cd37*^+^ confirmed this late increase, with significantly higher B cell density in surface fish compared to Pachón wounds, peaking at 30-dpci (Fig. 2j, k).

This data indicates that the prolonged immune response in surface fish is driven by macrophages and B cells, with macrophages dominating the intermediate stages and B cells playing a major role in later stages. While macrophages are known to be important for phagocytosis, cytokine production and collagen deposition during the early stages of fish heart regeneration, their roles in late-stage regeneration remain unexplored. Furthermore, the role of B cells in fish heart regeneration has not been characterised.

### Distinct functional profiles of late-stage immune cells

**Fig. 3:**
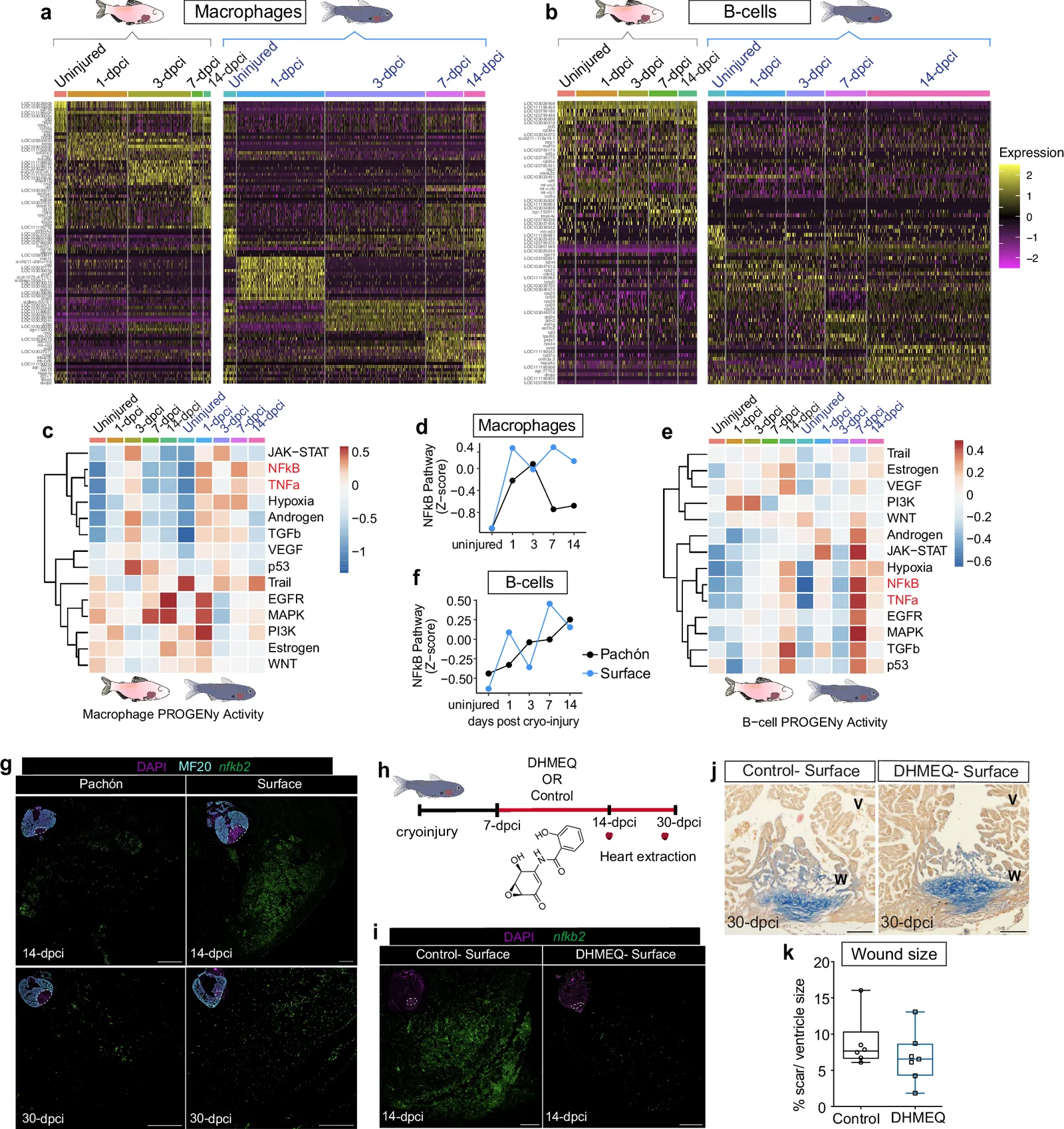
Distinct immune cell dynamics and pathway activation in surface fish and Pachόn at late stages after injury, with no direct impact of NF-κB inhibition on regeneration. Heatmaps displaying the top marker genes for macrophages (**a**) and B cells (**b**) across all timepoints in surface fish (left) and Pachón (right), shown at uninjured, 1-, 3-, 7- and 14- dpci. Dynamic gene expression patterns in surface fish contrast with the return to baseline observed in Pachόn by 7-dpci. **c**–**e** PROGENy pathway activity heatmaps for macrophages (**c**) and B cells (**e**) in surface fish and Pachόn across all timepoints. TNFα and NF-κB pathways (highlighted in red) are upregulated specifically at late stages in surface fish. Line plots showing NF-κB pathway activity (Z-score) over time in macrophages (**d**) and B cells (**f**) for Pachόn and surface fish, illustrating the sustained upregulation of NF-κB signalling at late stages in surface fish. **g** RNAscope revealing *nfkb2*^+^ cells (green) in Pachόn and surface fish ventricles at 14-dpci (top) and 30-dpci (bottom), counterstained with MF20 (cyan) and DAPI (magenta). Surface fish show strong NF-κB activation at both time points. The figure is representative of three independent experiments. **h** Experimental schematic: surface fish were injected with either Saline control or DHMEQ from 7- to 14-dpci and 7- to 30-dpci. Hearts were collected at 14-dpci and 30-dpci for RNAscope and AFOG staining, respectively. **i** RNAscope targeting *nfkb2* in control and DHMEQ-injected surface fish ventricles at 14-dpci, confirming effective inhibition of NF-κB signalling. **j** AFOG-stained images of control and DHMEQ-injected surface fish hearts at 30-dpci. **k** Quantification of the wound area in control and DHMEQ-injected hearts at 30-dpci shows no significant difference, Mann–Whitney *U* test, *p* = 0.366, *n* = 6,7. Boxplots show the median and the whiskers extending to the minimum and maximum values; individual points are overlaid. All scale bars, 100-μm. Source data for **k** is provided as a Source Data file. DHMEQ, dehydroxymethylepoxyquinomicin; dpci, days post cryo-injury; V, ventricle; W, wound.

To investigate whether the immune cells with prolonged presence in surface fish have specific functions promoting regeneration at late stages, we plotted the top markers for macrophages and B cells at uninjured, 1-, 3-, 7-and 14-dpci in both surface fish and Pachόn (Fig. 3a, b). This revealed dynamic transcriptional profiles over time in surface fish, with distinct gene sets activated at each time point, including late stages, indicating that the function of these cells changes over time with unique roles emerging during regeneration. In contrast, the transcriptional profiles of immune cells in Pachón largely returned to baseline by 7-dpci, reflecting a transient activation.

To explore the pathways uniquely activated in the late-stage leukocytes, we performed PROGENy pathway analysis^31^ on macrophages and B cells, revealing significant upregulation of TNFα and NF-κB signalling in both macrophages and B cells in surface fish at late stages (Fig. 3c–f). The late activation of TNFα and NF-κB in regenerating surface fish contradicts their conventional roles as pro-inflammatory signals that typically peak during the early stages of injury and are thought to inhibit regeneration when prolonged^32–34^. This finding highlights the possibility that, in a regenerative context, these pathways may serve a dual function, initially driving inflammation but later contributing to tissue remodelling and repair.

To validate the potential pro-regenerative role of NF-κB signalling, we first examined the presence of *nfkb2*^+^ cells in the ventricles of both surface and Pachón fish at 14- and 30-dpci using RNAscope. Surface fish exhibited strong NF-κB activation at both time points compared to Pachón (Fig. 3g). To further assess the functional impact of NF-κB at these late stages, we treated surface fish with dehydroxymethylepoxyquinomicin (DHMEQ), an NF-κB inhibitor, from 7- to 14-dpci or 30-dpci (Fig. 3h). RNAscope analysis confirmed a significant reduction in *nfkb2*^+^ cells in DHMEQ-treated surface fish at 14-dpci compared to controls, indicating effective inhibition of NF-κB signalling (Fig. 3i). However, there was no significant difference in wound size between DHMEQ-treated and control surface fish hearts at 30-dpci (Fig. 3j, k). These results suggest that although late-stage NF-κB activation is prominent in surface fish, its direct impact on cardiac regeneration may be limited.

### Late phagocytic macrophages are required for regeneration

**Fig. 4:**
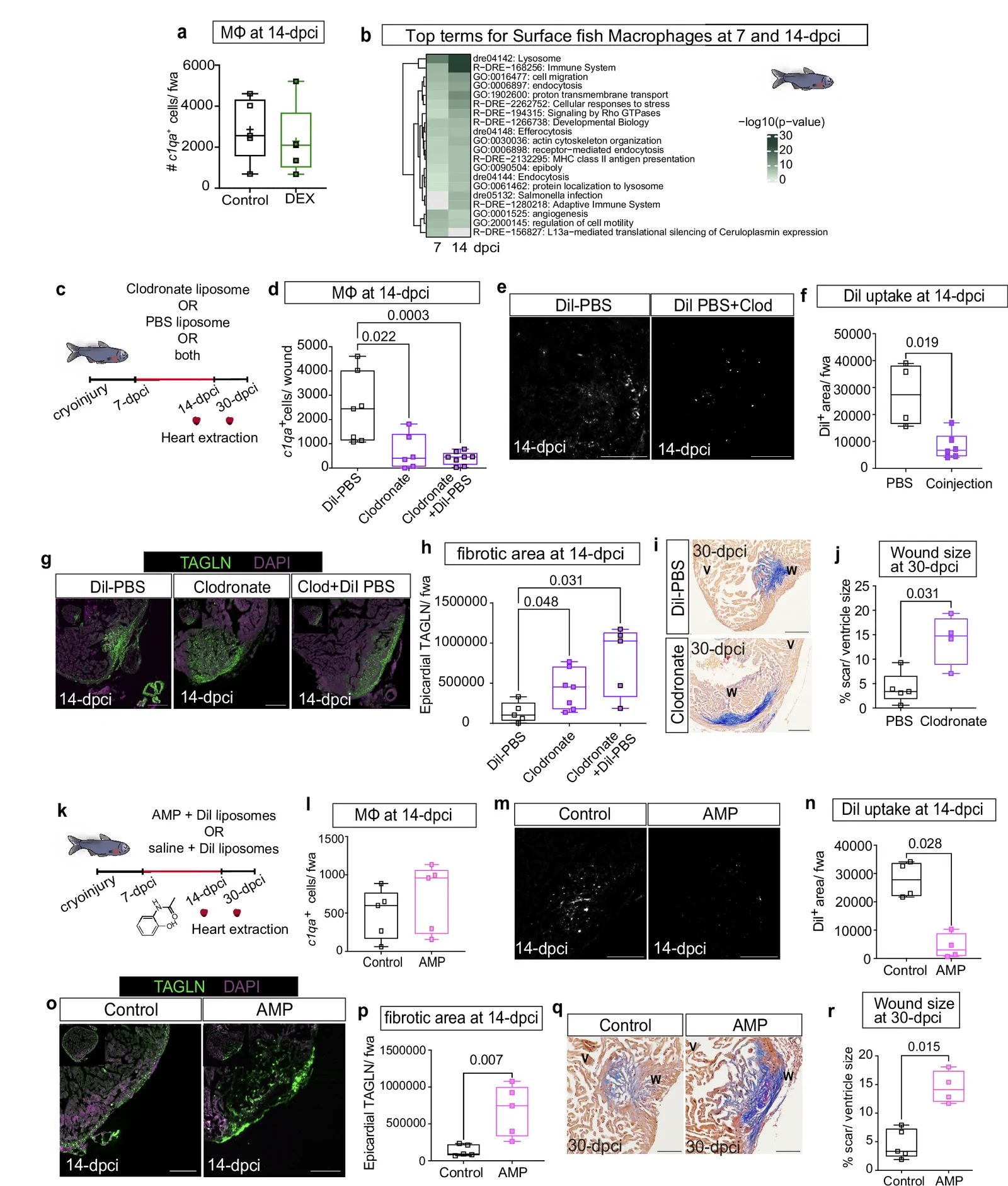
Late-stage macrophages are functionally active phagocytes essential for cardiac regeneration. **a** Quantification of *c1qa*^*+*^ macrophages at 14-dpci in Dex-treated and control surface fish hearts. Mann–Whitney *U* test, *p* = 0.42, *n* = 5 per group. **b** GO term analysis showing the top terms for surface fish macrophage marker genes at 7- and 14-dpci. **c** Experimental schematic: surface fish were injected with Clodronate liposomes, PBS liposomes or both between 7 and 14-dpci. **d** Quantification of *c1qa*^+^ macrophages in the wound at 14-dpci. two-sided Mann–Whitney *U* test; *p* = 0.017 (PBS vs Clodronate), *p* = 0.009 (PBS vs coinjection), *n* = 7,6, 8, respectively. **e** Representative images showing Dil liposome signal in Dil-PBS and co-injected hearts at 14-dpci. **f** Quantification of Dil^+^ area. Two-sided Mann–Whitney *U* test, *p* = 0.019; *n* = 4 PBS, and *n* = 6 coinjection. **g** Representative images of epicardial TAGLN (green) and DAPI (magenta) in DiI-PBS, Clodronate, and coinjection hearts at 14-dpci. **h** Quantification of epicardial TAGLN area at 14-dpci. Kruskal–Wallis test, *H* = 7.337, *p* = 0.026; pairwise Mann–Whitney *U* test, *p* = 0.048 (PBS vs Clodronate), *p* = 0.032 (PBS vs coinjection); *n* = 5,7, 5, respectively. All test performed were two-sided. **i** AFOG-stained sections of Dil-PBS and Clodronate-injected hearts at 30-dpci. **j** Quantification of the wound area as a percentage at 30-dpci. Two-sided Mann–Whitney *U* test, *p* = 0.031, *n* = 5 PBS, *n* = 4 Clodronate. **k** Experimental schematic: surface fish were co-injected Dil liposomes with either AMP or control between 7 and 14-dpci. **l** Quantification of *c1qa*^+^ macrophages at 14-dpci in control and AMP-treated hearts, two-sided Mann–Whitney-*U* test, *p* = 0.309; *n* = 5 per group. **m** Representative images showing Dil liposome signal in control and AMP-treated hearts at 14-dpci. **n** Dil^+^ area, two-sided Mann–Whitney *U* test, *p* = 0.029; *n* = 4 per group. **o** Representative images showing epicardial TAGLN (green) and DAPI (magenta) in control and AMP-treated hearts at 14-dpci. **p** Epicardial TAGLN area at 14-dpci, two-sided Mann–Whitney *U* test, *p* = 0.007; *n* = 4 per group. **q** AFOG-stained sections of control and AMP-treated hearts at 30-dpci. **r** The wound area as a percentage at 30-dpci. Two-sided Mann–Whitney *U* test, *p* = 0.015; control *n* = 5, AMP *n* = 4. All boxplots show the median with the minimum and maximum values. All scale bars, 100-μm. Source data for **a**, **d**, **f**, **h**, **j**, **l**, **n**, **p**, **r** are provided as a Source Data file. AMP, 2-acetamidophenol; dpci, days post cryoinjury; fwa, fractional wound area; TAGLN, transgelin; V, ventricle; W, wound.

Since surface fish exhibit a prolonged presence of functionally distinct immune cells at late stages of healing, we further examined the functions of the macrophages during this period. Quantification of *c1qa*^+^ macrophages in surface fish hearts at 14-dpci following Dexamethasone treatment revealed no significant difference in *c1qa*^+^ cell numbers between treatment and control hearts (Fig. 4a and Supplementary Fig. 3a), suggesting that macrophage persistence at late stages is regulated independently of broad inflammatory signalling. Gene Ontology (GO) term analysis of the top cell markers for surface fish macrophages at 7- and 14-dpci revealed *lysosome* as the top activated pathway at these late stages, alongside pathways related to cell migration and endocytosis (Fig. 4b). Given that scar tissue begins to visibly reduce from around 7-dpci, we next investigated whether these macrophages are actively phagocytic. Surface fish were injected with fluorescently labelled DiI PBS liposomes from 7- to 14-dpci, and hearts were harvested at 14-dpci. The majority of *c1qa*^+^ macrophages had ingested Dil liposomes (Supplementary Fig. 3c, d), confirming that these cells are actively phagocytic at late stages of injury.

To assess the functional importance of phagocytic macrophages, we depleted them using Clodronate liposomes from 7- to 14-dpci (Fig. 4c). Clodronate liposomes selectively deplete macrophages by inducing apoptosis in phagocytic cells upon uptake. Clodronate treatment significantly reduced *c1qa*^+^ macrophage numbers in the wound compared to Dil-PBS controls (Fig. 4d and Supplementary Fig. 3b). To confirm that Clodronate preferentially depletes phagocytic macrophages, we co-injected Clodronate with Dil liposomes and found significantly reduced Dil uptake compared to PBS-injected controls (Fig. 4e, f). To assess whether phagocytic activity contributes to scar clearance, we quantified epicardial transgelin (TAGLN) as a marker of fibrotic tissue at 14-dpci. Both Clodronate and Clodronate+Dil PBS co-injected hearts exhibited significantly elevated epicardial TAGLN levels compared to Dil-PBS controls (Fig. 4g, h), indicating impaired scar removal in the absence of phagocytic macrophages. Consistent with this, Clodronate-treated hearts exhibited significantly larger wounds at 30-dpci compared to controls (Fig. 4i, j), demonstrating that late phagocytic macrophages are essential for regeneration.

Additionally, as B cells can exhibit phagocytic activity in some contexts^35–37^, we only observed colocalisation specifically with *c1qa*-expressing cells, further supporting the idea that macrophages are the primary phagocytes in this context. Clodronate treatment did not affect B cell numbers in the wound (Supplementary Fig. 3h), suggesting that macrophages are not required for B cell recruitment. Furthermore, quantification of B cell numbers in the Panther zebrafish mutant, which lacks *csf1ra* macrophage populations, revealed no difference compared to wild-type controls at 14-dpci (Supplementary Fig. 3i, j). Together, this shows that B cell recruitment during cardiac repair is independent of macrophage presence.

To directly test whether phagocytosis is the primary mechanism by which late-stage macrophages support regeneration, we treated surface fish with 2-acetamidophenol (AMP), a pharmacological inhibitor of phagocytosis, co-injected with Dil liposomes from 7- to 14-dpci (Fig. 4k). AMP treatment did not affect macrophage numbers in the wound (Fig. 4l), confirming that the treatment specifically targets phagocytic function rather than macrophage survival. AMP-treated fish showed a significant reduction in Dil liposome uptake compared the controls (Fig. 4m, n), confirming effective inhibition of phagocytosis in vivo. Consistent with the Clodronate results, AMP-treated hearts exhibited significantly elevated epicardial TAGLN levels at 14-dpci (Fig. 4o, p) and significantly larger wounds at 30-dpci (Fig. 4r, s), demonstrating that phagocytosis is the primary mechanism through which late-stage macrophages support cardiac regeneration and scar clearance^11,30–32^.

### B cells promote cardiomyocyte cell cycle progression

**Fig. 5:**
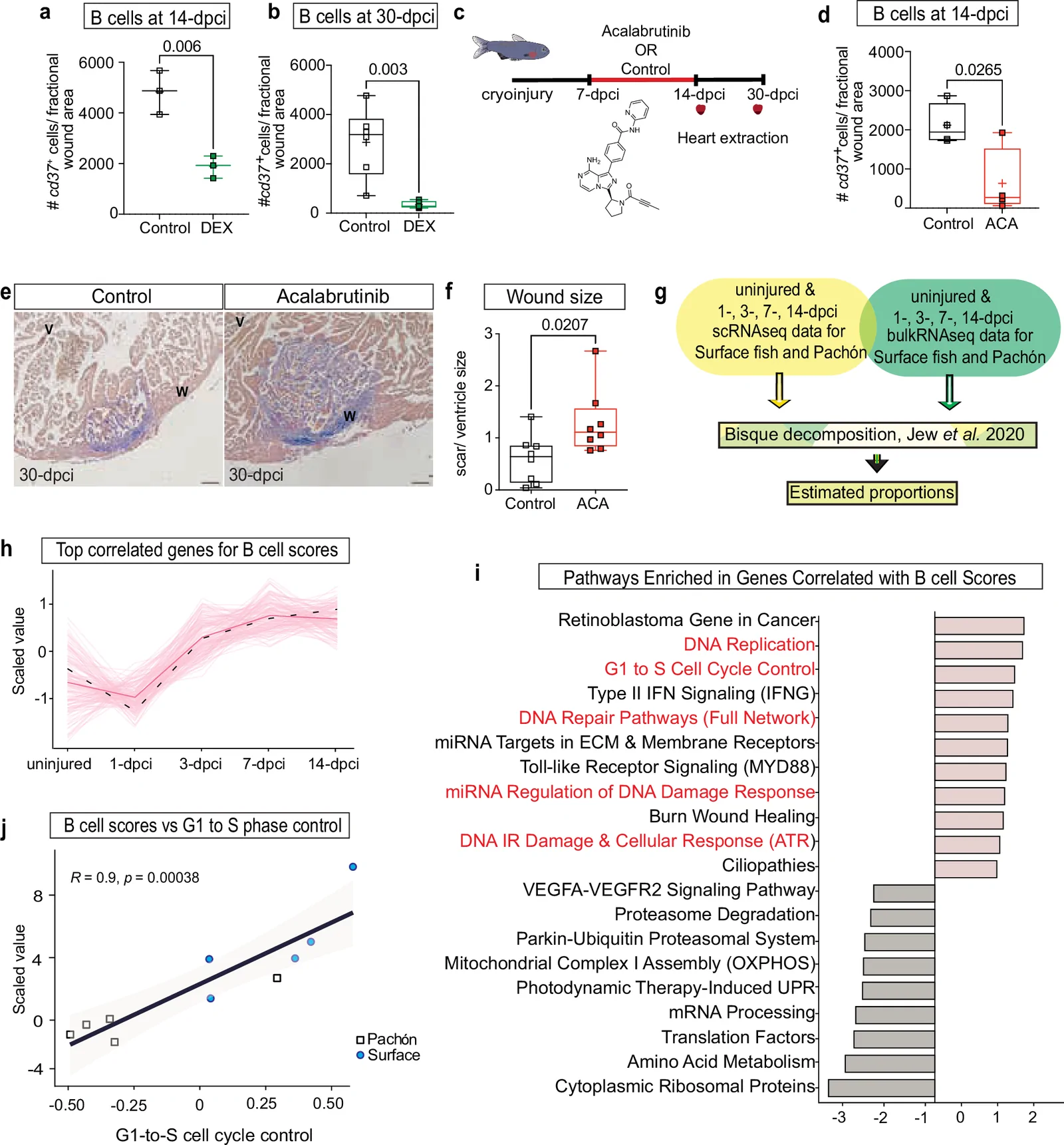
Temporal dynamics of B cells and their involvement in cell cycle regulation during cardiac regeneration in surface and Pachón fish. Quantification of *cd37*^*+*^ B cells at 14-dpci (**a**), and at 30-dpci (**b**) in Dexamethasone-treated and control surface fish hearts show significantly impaired B cell response upon Dexamethasone treatment in both time points, two-sided Student’s *t* test, *n* = 3–6 each group *p* = 0.006 and *p* = 0.003, respectively. Data are presented as individual values indicating the median and error bars spanning the minimum and maximum values. **c** Experimental schematic: surface fish were injected with either control or Acalabrutinib from 7- to 11-dpci. Hearts were collected at 14-dpci and 30-dpci for RNAscope and AFOG staining. **d** Quantification of *cd37*^+^ B cells present in the wound at 14-dpci in control vs Acalabrutinib-injected surface fish hearts, showing significantly reduced B cell numbers upon inhibitor treatment. Two-sided Student’s *t* test, *p* = 0.027; *n* = 4 per group. **e** AFOG-stained sections of control and Acalabrutinib-injected surface fish hearts at 30-dpci, showing larger scars in the Acalabrutinib group. Scale bar 100-μm. **f** Quantification of wound area at 30-dpci, showing significantly larger scars in Acalabrutinib-treated fish compared to controls. Two-sided Mann–Whitney *U* test, *p* = 0.021, *n* = 8 per group. Boxplots show the median and the whiskers extending to the minimum and maximum values; individual points are overlaid. **g** Immune deconvolution workflow: single-cell RNAseq and bulk RNAseq data from surface fish and Pachόn at uninjured, 1-, 3-, 7- and 14-dpci were integrated using Bisque decomposition to estimate cell-type proportions. **h** Temporal dynamics of the top genes correlated with B cell deconvolution scores in surface fish, showing increased B cell-associated gene expression starting at 7-dpci. **i** WikiPathways enrichment analysis of genes positively correlated with B cell scores in bulk RNAseq data from surface fish, highlighting significant upregulation of proliferation, cell cycle regulation and DNA repair pathways (shown in red). **j** Correlation between B cell deconvolution scores and G1-to-S phase cell cycle control pathway scores across surface fish and Pachόn samples at all time points, showing strong positive correlation (*R* = 0.9, *p* = 0.00038, two-sided), indicating that B cell activity is associated with cell cycle progression. Source data for **a**, **b**, **d**, **f** are provided as a Source Data file. ACA, Acalabrutinib; DEX, Dexamethasone; dpci, days post injury; NES, normalised enrichment score.

Following the demonstration that late-stage macrophages are critical for cardiac regeneration through their phagocytic activity, we sought to explore the role of B cells. To investigate the contribution of B cells to regeneration, we analysed *cd37*^+^ B cells in surface fish hearts at 14- and 30-dpci following Dexamethasone treatment. We observed a significant reduction in *cd37*^+^ cell numbers at both 14- and 30-dpci compared with controls (Fig. 5a, b), indicating that, in contrast to late macrophages, B cell recruitment relies on pro-inflammatory signals.

To dissect the role of B cells, surface fish were treated with Acalabrutinib, a Bruton tyrosine kinase (BTK) inhibitor that specifically targets B cells, from 7- to 11-dpci, and hearts were analysed at 14- and 30-dpci (Fig. 5c). Acalabrutinib treatment significantly reduced *cd37*^+^ B cell numbers in the wound area at 14-dpci compared to controls (Fig. 5d). At 30-dpci, Acalabrutinib-treated hearts exhibited significantly larger wounds compared to controls, demonstrating that B cell depletion impairs regeneration (Fig. 5e, f). To determine the critical window of B cell activity, we treated fish with Acalabrutinib from 2- to 5-dpci, prior to B cell recruitment, or from 26- to 30-dpci, during late B cell activity, Neither treatment window produced a significant effect on wound size at 30-dpci (Supplementary Fig. 4c–g, h–l), confirming that the critical period of B cell activity coincides specifically with their recruitment window from 7-dpci onwards, Notably, Acalabrutinib treatment from 26- to 30-dpci showed a trend towards reduced B cell numbers and larger wounds (Supplementary Fig. 4j–l), suggesting that late B cell activity may still contribute to regeneration with a longer treatment.

Single-cell analysis of B cells was challenging due to their low number in Pachón cavefish and the high proportion of functionally unannotated genes in our dataset, particularly in these cells, making it difficult to investigate their functions further using this data. To enable more comprehensive immune profiling of B cells, we used deconvolution to leverage our scRNA-seq data to estimate cell-type composition and abundance in bulk RNA-sequencing, which provides greater depth and sample size^38^. We performed immune deconvolution analysis on bulk RNA-seq data from Pachón and surface fish hearts at multiple time points (uninjured, 1-, 3-, 7- and 14-dpci)^39^ (Fig. 5g). We first validated our differential proportion analysis for the major immune cell types and observed the expected early immune response dynamics. Neutrophils showed a rapid induction, peaking at 1-dpci, followed by a peak in macrophages at 3-dpci and a prolonged elevated response, consistent with their trends observed in the earlier DPA results (Fig. 2 and Supplementary Fig. 4m, n).

Consistent with our previous results, deconvoluted B cell scores were significantly elevated in surface fish compared with Pachón at 14-dpci (Supplementary Fig. 4o). To elucidate the processes associated with B cell activity in surface fish, we identified genes that were correlated with B cell scores across the injury time course, which increased at 7-dpci and were sustained at 14-dpci (Fig. 5h). Several pathways involved in proliferation, regulation of the cell cycle, and wound healing were significantly enriched in the positively correlated genes (Fig. 5i). Notably, deconvoluted B cell scores correlated robustly with GSVA summary scores for the G1-to-S phase cell cycle control pathway in surface and Pachón cavefish samples at 14-dpci (Fig. 5j). Therefore, B cells appear to be closely associated with overall proliferation rates in the heart. While cardiomyocyte proliferation is a critical driver of successful cardiac regeneration^1,10^, it remained unclear whether B cells themselves could proliferate. To determine whether the observed correlation was due to B cell proliferation, we examined B cell-specific cell cycle activity. Analysis of scRNA-seq data for *pcna* expression in B cells revealed no significant activation of proliferation in B cells from 7-dpci onwards (Supplementary Fig. 4p). As the B cells did not proliferate, the correlation between deconvoluted B cell scores and cell cycle pathways suggests that B cells may facilitate cardiomyocyte proliferation through paracrine mechanisms.

### B cells regulate border zone cardiomyocyte proliferation

**Fig. 6:**
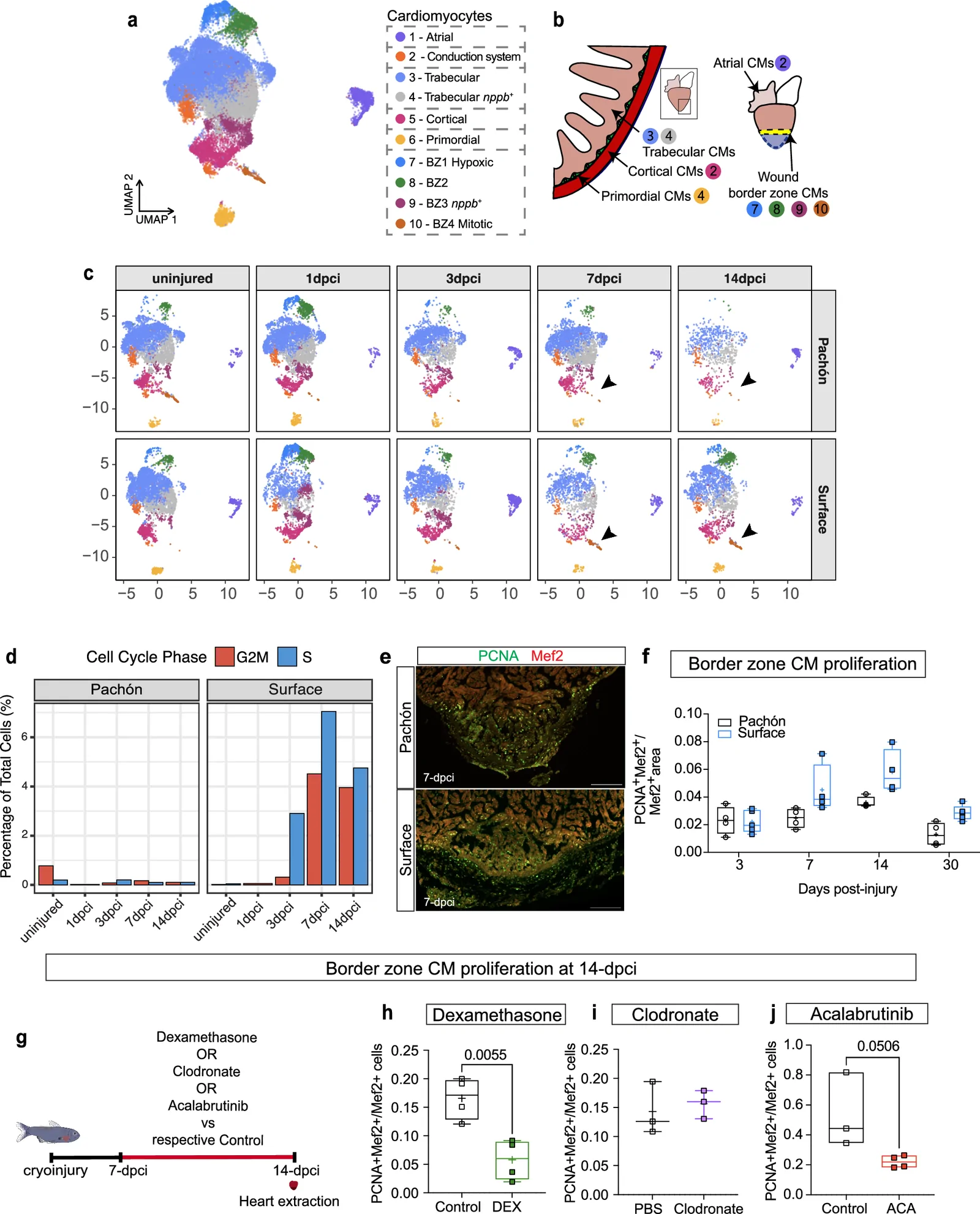
Border zone cardiomyocyte proliferation is disrupted in Pachόn due to negative regulation of the cell cycle. **a** UMAP of re-clustered cardiomyocyte scRNA-seq data showing ten sub-populations: atrial (1), conduction system (2), trabecular (3), trabecular *nppb*^+^ (4), cortical (5), primordial (6) and four wound border zone clusters; BZ1 Hypoxic (7), BZ2 (8), BZ3 *nppb*^+^ (9) and BZ4 Mitotic (10). **b** Schematic illustrating the anatomical location of each cardiomyocyte sub-population within the heart, with border zone clusters (7–10) localised to the wound region. **c** UMAPs showing cardiomyocyte sub-populations over time in Pachόn (top) and surface fish (bottom) at uninjured, 1-, 3-, 7- and 14-dpci. Arrowheads indicate the emergence of border zone clusters after injury. **d** Bar charts showing the percentage of total cardiomyocytes in S-phase (blue) and G2M-phase (red) at each time point in Pachόn (left) and surface fish (right), demonstrating a progressive increase in cycling cardiomyocytes in surface fish from 3-dpci onwards that is absent in Pachόn. **e** Representative images showing PCNA (green) and Mef2 (red) in Pachόn (top) and surface fish (bottom) ventricles at 7-dpci. Scale bar 100-μm. **f** Quantification of PCNA^+^Mef2^+^ border zone cardiomyocytes normalised to Mef2^+^ area at 3-, 7-, 14- and 30-dpci in Pachόn and surface fish. Two-way ANOVA with Bonferroni post-hoc correction. Main effect of morph: *F*(1, 26) = 15.14, *p* = 0.0006; main effect of timepoint: *F*(3, 26) = 12.01, *p* < 0.0001; interaction: *F*(3, 26) = 2.21, *p* = 0.110. Pachόn *n* = 4 (3-, 7-, 14- and 30-dpci), Surface *n* = 5 (3- and 30-dpci), *n* = 4 (7- and 14-dpci). **g** Experimental schematic: surface fish were treated with Dexamethasone, Clodronate liposomes or Acalabrutinib versus their respective controls from 7- to 14-dpci. **h** Quantification of PCNA^+^Mef2^+^ border zone cardiomyocytes at 14-dpci in Dexamethasone-treated and control hearts. Two-sided Student’s *t* test, *p* = 0.006; *n* = 4 per group. **i** Quantification of PCNA^+^Mef2^+^ border zone cardiomyocytes at 14-dpci in Clodronate-treated and PBS control hearts. Unpaired t-test, *p* = 0.675; *n* = 3 per group. Data are presented as individual values indicating the median and error bars spanning the minimum and maximum values. **j** Quantification of PCNA^+^Mef2^+^ border zone cardiomyocytes at 14-dpci in Acalabrutinib-treated and control hearts. Two-sided Student’s *t* test, *p* = 0.051; control *n* = 3, ACA *n* = 4. Source data for **f**, **h**, **i**, **j** is provided as a Source Data file. ACA, Acalabrutinib; BZ CM, border zone cardiomyocytes; DEX, Dexamethasone; dpci, days post injury.

Given that cardiomyocytes are the most abundant cell type in the heart, our bulk data may reflect a relationship between B cell activity and cardiomyocyte proliferation^19^. To investigate this, we assessed border zone cardiomyocyte behaviour in surface fish and Pachón before and after cryoinjury by subsetting the scRNA-seq data to isolate the cardiomyocytes based on *myl7* expression (Supplementary Fig. 5a). Re-clustering revealed ten cardiomyocyte sub-populations, including atrial, trabecular *nppb*^+^, cortical and primordial cardiomyocytes present during homoeostasis, alongside four wound border zone clusters emerging after cryoinjury (Fig. 6a, b and Supplementary Fig. 5b).

In both surface fish and Pachón, border zone cardiomyocyte clusters appeared after injury. Border zone clusters BZ1, 2 and 3 emerged at 1-dpci, whereas BZ cluster 4 appeared from 3-dpci. BZ cluster 4 was characterised by M-phase activity (Fig. 6a–c). All BZ clusters, including the mitotic BZ4 cluster, remained highly active in surface fish at 14-dpci, coinciding with the period when B-cell numbers have started to increase, further suggesting a correlation between the prolonged immune response and cardiomyocyte proliferation. Clear differences in border zone clusters between Pachon and surface fish became apparent, particularly at 14-dpci, with a strong reduction in BZ clusters 1–3 in Pachόn compared to surface fish. Mitotic BZ cluster 4 was markedly reduced in Pachόn at all timepoints after injury.

Analysis of cell cycle parameters showed that S-phase was initiated at 3-dpci in surface fish, peaked at 7-dpci with sustained high levels at 14-dpci, while M-phase was initiated slightly later, with similar levels at 7- and 14-dpci (Fig. 6d). In contrast, both S- and M-phase levels remained close to baseline in Pachόn throughout the time course, consistent with the absence of B cell response in this morph.

This complete lack of cell cycle progression in Pachόn was surprising, as we have previously shown that cardiomyocyte proliferation, as measured using a PCNA antibody in border zone cardiomyocytes, was induced in both Pachón and surface fish after resection injury^19^. We therefore repeated this analysis using PCNA and Mef2 immunostaining on sections (Fig. 6e). This recapitulated the insignificantly higher levels of *pcna* expression in Surface fish seen after resection injury, with proliferation remaining significantly elevated for longer at 30-dpci, reflecting the longer period of time required to complete regeneration after cryoinjury compared to resection injury (Fig. 6f). However, a discrepancy exists between the single cell mRNA level cell cycle data and PCNA protein levels, with Pachόn showing similar PCNA protein levels to surface fish despite the absence of M-phase progression. As previously observed after resection injury, a possible explanation for this discrepancy is that Pachόn cardiomyocytes become arrested in S-phase, retaining PCNA protein expression without progressing to mitosis, as also supported by ploidy analysis in these fish^40^. In surface fish, cardiomyocytes progress through the full cell cycle, generate new cardiomyocytes, and likely upregulate *pcna* mRNA again to initiate subsequent cell cycles.

To understand whether late cardiomyocyte proliferation is regulated by the prolonged immune response, we analysed border zone cardiomyocyte proliferation in surface fish hearts treated with Dexamethasone, Clodronate or Acalabrutinib between the days 7- and 14-dpci (Fig. 6g). Dexamethasone treatment significantly reduced border zone cardiomyocyte proliferation at 14-dpci, while Clodronate had no significant effect, and Acalabrutinib resulted in a marked reduction in proliferating border zone cardiomyocytes (Fig. 6h–j). This suggests that the B cell response, rather than macrophage phagocytic activity, is the primary immune driver of cardiomyocyte proliferation during late-stage regeneration. To exclude the possibility of off-target effects of Acalabrutinib directly affecting cardiomyocyte proliferation, we injected fish from 2- to 5-dpci, prior to B cell recruitment and isolated the hearts at both 5- and 30-dpci. No difference in border zone cardiomyocyte proliferation was observed at either time point (Supplementary Fig. 5c–e), confirming that the effect of Acalabrutinib on cardiomyocyte proliferation is specifically mediated through B cell inhibition during their recruitment window, and that B cells are critical for supporting cardiomyocyte proliferation and efficient wound healing during cardiac regeneration.

### Role of B cells in regeneration across species

**Fig. 7:**
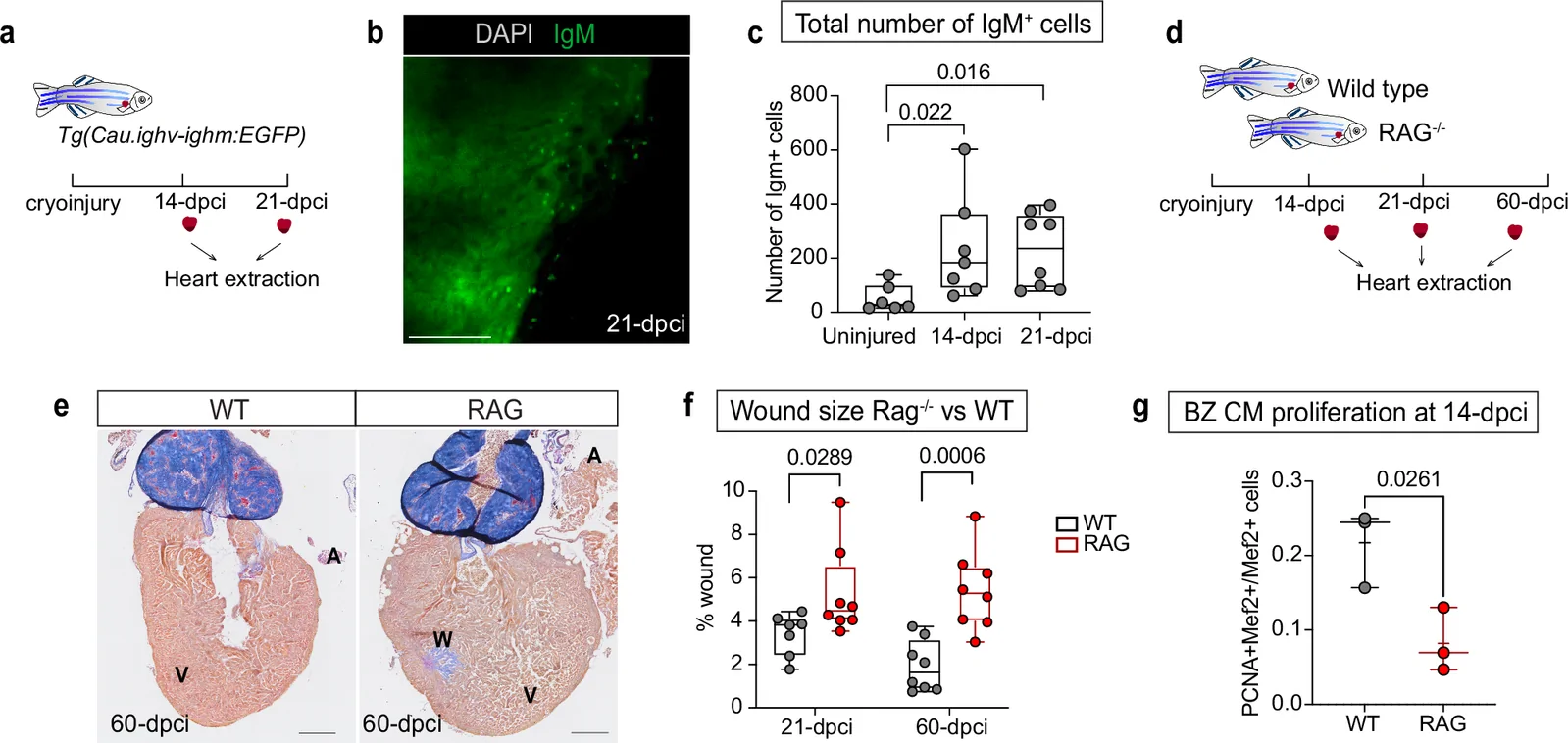
Cross-species validation of B cell involvement in cardiac regeneration. **a** Schematic indicating the timeline of zebrafish IgM experiments. **b** Representative image of 21-dpci Tg(*Cau.ighv-ighm:EGFP*)^42^ zebrafish heart showing IgM cells in the wound zone. **c** Quantification of IgM^+^ cells in Tg(*Cau.ighv-ighm:EGFP*) hearts at 14- and 21-dpci as well as unwounded controls reveals a significantly elevated IgM response at 21-dpci, two-sided Mann–Whitney *U* test; uninjured *n* = 6, 14-dpci *n* = 7, and 21-dpci *n* = 8. **d** Wild-type and *rag1*^-/-^ zebrafish were cryoinjured, and the hearts were collected at 14-, 21- and 60-dpci for RNAscope, immunostainings and AFOG staining. **e** Representative images of wild-type and *rag1*^-/-^ zebrafish hearts at 60-dpci, the wound area is indicated with a black dashed line. **f** Quantification of wound size of *rag1*^-/-^ and wild-type control hearts at 21- and 60-dpci shows significantly larger wounds in *rag1*^-/-^ mutant hearts, two-sided Mann–Whitney *U* tests. *n* = 7 (21-dpci WT), *n* = 8 (60-dpci WT, 21- and 60-dpci RAG). **g** Quantification of Mef2/ PCNA double-positive cells show significant difference between wild-type control vs *rag1*^-/-^ zebrafish for the proliferating cardiomyocytes at the border zone, two-sided Mann–Whitney *U* tests, *n* = 3/each group. Data are presented as individual values indicating the median and error bars spanning the minimum and maximum values. All scale bars, 100-μm. Source data for **c**, **f**, **g** are provided as a Source Data file. W: wound, A: atrium, V: ventricle; WT, wild type, dpci, days-post-injury.

To further validate our findings in a different species, we turned to the zebrafish model, which shares many regenerative features with surface fish^41^. We analysed IgM^+^ cell populations in adult zebrafish hearts following cryoinjury using Tg(*Cau.ighv-ighm:EGFP*)^42^ zebrafish (Fig. 7a). IgM, which is labelled with EGFP in this line, is the major immunoglobulin and a well-established marker for the predominant subset of B cells in zebrafish. Quantification of EGFP-positive cells revealed a significant increase in IgM^+^ cells in hearts at 14- and 21-dpci compared to unwounded controls (Fig. 7b, c). To test the functional role of B cells in zebrafish regeneration, we used *rag1*^*-/-*^ mutant zebrafish, which lack mature lymphocytes, including B cells^43,44^. At 21- and 60-dpci, wild-type hearts exhibited robust regeneration, while *rag1*^*-/-*^ hearts showed impaired repair, as evidenced by quantification of wound size at 21- and 60-dpci (Fig. 7d, e). Similar to the observations in surface fish, we found a significant reduction in proliferating cardiomyocytes at the border zone in *rag1*^*-/-*^ hearts compared to wild-type controls (Fig. 7f).

These findings indicate that the B cell response is conserved among different regenerative species and plays a critical role in promoting cardiomyocyte proliferation and efficient cardiac regeneration.

## Discussion

In this study, we leveraged scRNA-seq and immune profiling to identify key factors influencing the difference in cardiac regeneration between surface and Pachón subtypes of *A. mexicanus* and further verified our B cell findings in regenerative adult zebrafish. We observed a significantly diverging immune response between regenerative surface fish and scarring Pachón following cryoinjury. Surface fish exhibited a prolonged activation of macrophages and B cells, which were associated with successful cardiac repair, while Pachón showed a more transient immune response. These findings, showing that a prolonged immune response is crucial for successful regeneration, are surprising as extended inflammation is considered detrimental to regeneration.

Studies involving macrophage ablation in zebrafish and neonatal mice have focused on the early days after injury, showing that disrupting the initial macrophage influx inhibits regeneration^9,13,30,45^. Similarly, Medaka, another teleost fish species that exhibits limited cardiac regeneration, unlike zebrafish, completely lack the macrophage response^46^. While Pachόn also do not regenerate, their response differs significantly from Medaka, indicating key differences between fish species. Pachόn exhibits a remarkably similar initial influx of neutrophils and macrophages to regenerative surface fish, yet regeneration is still unsuccessful. It is only after the initial peak that significant differences, which are crucial for regeneration, appear in the immune response between the two morphs. We were able to pick up on these more subtle differences as *Astyanax mexicanus* provides the unique opportunity to directly compare between two morphs of the same species with different regenerative abilities, surface fish and Pachόn. This provides the granularity needed to identify fine nuances in the response that determine the difference between successful regeneration and scarring, which might be lost when comparing entirely different species or using blunt cell ablation approaches.

The beneficial role for a prolonged presence of macrophages during regeneration is surprising, especially as the cells upregulate pro-inflammatory genes such as *tnfa* and *nfkb2* specifically at these late stages. The prevailing consensus in the field is that a balance between pro-inflammatory and reparative gene expression in macrophages is key to regeneration and that prolonged pro-inflammatory activation of macrophages disrupts regeneration^47,48^. However, our findings indicate that while the late upregulation of pro-inflammatory genes does not positively contribute to regeneration, it also does not hinder it, as would be predicted based on previous studies^9,13^. NF-κB has been shown to be required for cardiomyocyte proliferation during regeneration^49^ but we did not find an effect on regeneration when inhibiting NF-κB during late stages, indicating the effect of NF-κB on cardiomyocyte proliferation occurs during the earlier stages of regeneration. Prolonged NF-κB and TNFα signatures might be key to maintaining specific cellular functions of macrophages throughout late stages of regeneration. For example, TNFα has been shown to enhance phagocytosis in macrophages by upregulating phagocytic receptors and stimulating reactive oxygen production^50,51^. Tissue-specific targeting of NF-κB and TNFα will be required to better understand their role at these late stages, as overall inhibition did not show effects on regeneration.

Phagocytosis, a key task of macrophages during early stages^9,30,52^, indeed appeared to be a crucial function of late-stage macrophages. Most macrophages present after 7-dpci were actively phagocytic, suggesting a role in wound clearance and tissue remodelling. It is only after 7-dpci that the wound strongly starts to reduce in size, but the mechanisms of wound clearance have not yet been established. Our use of two independent approaches with Clodronate liposome depletion and pharmacological inhibition of phagocytosis with 2-acetamidophenol demonstrated that it is specifically the phagocytic function of late-stage macrophages, rather than their physical presence, that is required for scar clearance and regeneration. Both approaches resulted in elevated epicardial transgelin levels at 14-dpci and significantly larger wounds at 30-dpci, consistent with impaired scar resolution. These findings are further supported by a recent study in zebrafish demonstrating that border zone cardiomyocytes and macrophages work in close physical contact to regulate ECM remodelling, with macrophages being essential for collagen degradation and subsequent cardiomyocyte protrusion into the injured tissue via mmp14b-dependent mechanism^53^. Our GO term analysis of late-stage surface fish macrophages showing enrichment of lysosomal, efferocytosis and endocytosis pathways is entirely consistent with this picture of macrophages as active mediators of ECM clearance rather than inflammatory signalling at late stages. Clodronate treatment also enhanced fibroblast proliferation in the wound, suggesting that a lack of clearance allows fibroblasts to settle and initiate proliferation to establish a permanent scar, although direct signalling mechanisms may also contribute^11,54,55^.

The reduced phagocytic capacity of Pachόn is also noteworthy. Peuß et al. demonstrated that innate cellular immune responses, including phagocytosis, are drastically decreased in cavefish compared to surface fish^22^. The inability of Pachόn macrophages to sustain phagocytic activity at late stages may therefore represent a key mechanistic bottleneck preventing scar clearance and regeneration in this morph.

While our scRNA-seq data suggested a prolonged initial peak in neutrophil influx in Pachón compared to surface fish, as well as low-level retention in surface fish after 3-dpci compared to Pachόn, neither observation was confirmed in tissue sections. Neutrophils are known to have dual roles in cardiac injury. A prolonged initial influx, as seen in the single-cell data in Pachón, has been strongly linked to scarring in the mammalian heart^56^, while certain pro-regenerative neutrophil states have also been described^57–59^. Although neutrophils may not be the primary drivers of the differences in regeneration between surface fish and Pachόn, their role in the early inflammatory response and potential contribution to tissue repair needs further elucidation.

In contrast to neutrophils, we confirmed B cells as a critical cell type involved in late-stage regeneration. Large numbers of B cells were recruited in both surface fish and zebrafish during late stages of regeneration. Inhibition of B cells using Acalabrutinib in surface fish or through mutation of *rag1* in zebrafish resulted in reduced cardiomyocyte proliferation and regeneration, indicating the positive influence of B cells on regeneration is conserved between species. While RAG mutants lack mature B and T cells due to defective V(D)J recombination, a critical process for adaptive cell development^44,60^, our data suggest that the effect on regeneration is due to B cell loss, as T cells are not prominently involved in late stages of cardiac regeneration and their numbers remain low. Our findings fit well with recent findings from neonatal mice^17^, in which depletion of B cells was found to reduce cardiomyocyte proliferation and impair cardiac function. Importantly, the same study found that adult B cells in mice promote inflammation and impair cardiac function after MI, suggesting that the pro-regenerative role of B cells is context- and developmental stage-dependent.

Regulatory B cells (Bregs) have also been shown to improve ventricular remodelling after MI by modulating monocyte migration and reducing inflammation^61^. Secretion of IL-10 by Bregs creates a pro-regenerative environment that may indirectly support cardiomyocyte proliferation by reducing inflammation and fibrosis, with a similar function found for bone marrow-derived naïve B cells^62^. This suggests that B cells play a conserved role in supporting cardiac repair across different injury models, although we could not find conserved mechanisms, as IL-10, for example, was not highly expressed in surface fish B cells. In non-mammalian models, B cells have not yet been implicated in tissue regeneration, and it has long been suggested that there is an inverse relationship between levels of adaptive immune response and ability to regenerate^11,63^. However, our findings indicate that a well-established adaptive B cell response, particularly in surface fish, supports the regenerative process.

The effect of B cells on cardiomyocyte proliferation is somewhat unexpected, as proliferation is initiated from 3-dpci, while B cell invasion only takes place from 7-dpci onwards. Our cell cycle analysis showed that border zone cardiomyocytes in surface fish progress from S-phase at 3 to 7-dpci to M-phase from 7-dpci onwards, and B cells arrive precisely at this transition point, suggesting they promote cell cycle progression into mitosis rather than initiating DNA replication. Inhibiting B cell signalling before their recruitment (2–5 dpci) had no effect on proliferation, whereas inhibition during the active B cell infiltration period, from 7- to 14-dpci, significantly reduced it. It is specifically the presence and activity of B cells that drives cardiomyocyte cell cycle progression into M-phase. IL-10, which is secreted by mammalian B cells, is a cytokine that has been shown to specifically affect cell cycle progression into M-phase in tendon-derived stem cells, mediated through the JAK/Stat3 pathway^64^. A similar mechanism could apply to fish cardiomyocytes, where cytokines secreted by B cells may drive cell cycle progression into mitosis and subsequent cytokinesis, an essential part of successful regeneration, which does not occur in their mammalian equivalents^65^. Interestingly, JAK/STAT signalling has independently been shown to promote cardiomyocyte proliferation, and this pathway may represent a convergent mechanism linking immune signals to cardiomyocyte cell cycle control^66,67^. Although IL-10 was not highly expressed in surface fish B cells in our dataset, the convergence of these pathways warrants future investigation into whether analogous cytokine-mediated mechanisms operate in teleost cardiac B cells.

Our findings identify macrophage phagocytosis to clear fibrotic scar tissue and B-cell-mediated promotion of cardiomyocyte proliferation as two distinct immune mechanisms that could serve as therapeutic targets to promote cardiac repair. These mechanisms operate sequentially and independently, suggesting that combinatorial approaches targeting both could be more efficient than single-pathway interventions. A recent study in medaka demonstrated that immune modulation promoting debris clearance via macrophage activity, combined with Granulin-mediated stimulation of cardiomyocyte proliferation, was sufficient to promote regenerative healing^68^, providing proof of concept for this combinatorial immune-regenerative strategy.

As the prolonged immune cell response was absent in Pachón, which has a strongly altered metabolism^69,70^, this raises the possibility that immune cell populations in Pachón do not have the energy to sustain a long-term response as a result of a metabolic trade-off favouring survival over costly immune functions. Pachόn cavefish exhibit elevated blood glucose, insulin resistance and hypertrophic visceral adipocytes compared to surface fish^71^, reflecting profound metabolic remodelling as an adaptation to cave life. In resource-limited environments, species often prioritise innate immunity over adaptive immunity due to lower energetic costs of innate defences, as seen in birds and rodents^72,73^. In the case of Pachόn, the loss of sustained B cell activity and other immune functions may reflect a similar trade-off, where adaptation to nutrient poor cave environment comes at the expense of adaptive immune responses^74,75^.

In conclusion, our study demonstrates that a prolonged immune response, driven by sustained activation of macrophages and B cells, is critical for successful cardiac regeneration. Late-stage macrophages promote regeneration through phagocytic clearance of fibrotic scar tissue, while B cells support cardiomyocyte cell cycle progression into mitosis through paracrine mechanisms yet to be fully elucidated. The transient immune response in Pachόn is characterised by insufficient immune activity, reduced phagocytic capacity, and absence of a B cell response; deficits that are likely rooted in the metabolic constraints of cave adaptation. These findings provide a framework for understanding the complex interplay between immune cells and tissue repair, with potential implications for improving regenerative strategies in mammals, including humans.

## Methods

### Animal husbandry

All *Astyanax mexicanus* procedures were conducted at the aquatic animal facilities of Biomedical Services and the Institute of Developmental and Regenerative Medicine at the University of Oxford. These procedures comply with the revised UK Animals (Scientific Procedures) Act 1986, Directive 2010/63/EU of the European Parliament and the Council of 22 September 2010 on the protection of animals used for scientific purposes, Home Office licenses, and were approved by Oxford University’s central Committee on Animal Care and Ethical Review (ACER).

All *Astyanax mexicanus* experiments were performed using wild-type surface fish from the Río Choy background and cavefish from the Pachón cave background. Fish were maintained in system water at 21–23 °C under a 14:10-h light/dark cycle. Adults of 1–2 years if age were used consistently. Sex was not formally assigned for every sample, as reliable sex determination methods are not optimised for *A. mexicanus*; however, mixed-sex cohorts were used to minimise potential bias.

*Danio rerio* (zebrafish) experiments were conducted at the University of Bristol in accordance with UK Home Office and by the local University of Bristol Animal Welfare and Ethical Review Body (AWERB). The Tg(*Cau.ighv-ighm:EGFP*)^42^ and *rag1*^*hu199*^^43^ mutants have been described previously and were maintained under standard conditions. Age matched wild-type fish served as controls for all *rag1*^-/-^ experiments.

### Cardiac injury

Cryoinjury was performed as the preferred method of cardiac injury, largely following a previously described protocol for zebrafish^76^. Briefly, fish were anesthetised in 0.25 g/L MS222 and transferred to a wet sponge. Scales on the thorax were removed with forceps, and a small incision was made using spring scissors. The pericardium was carefully removed, and the heart surface was dried using sterile lint-free tissue. Cryoinjury in *A. mexicanus* was performed inside the chest cavity, whereas in zebrafish, it was applied to the surface of the incision as the heart elevates when slight pressure is applied to the abdomen. A copper cryoprobe (0.5 cm diameter) was cooled in liquid nitrogen for a few seconds and placed on the ventricle until thawed. Injury size was monitored by observing colour changes in the area, and recovery was completed within 1–2 min by pipetting water over the gills.

Surface fish were individually housed post-surgery to minimise stress responses. For drug treatments, fish were randomly assigned to treatment groups at the end of the operation. Sham surgeries involved making a thorax incision, removing the pericardial sac, and placing a room-temperature copper probe on the ventricle before returning the fish to system water. All procedures were conducted at the same time of day by the same individual to minimise variability. Control hearts were isolated from uninjured fish, while the sham group hearts were isolated 3 days post-operation. Cryoinjured hearts were isolated at various time points between 1 and 60 days for cryoinjury.

### Single-cell RNA sequencing and analysis

Ventricles were isolated, digested, and processed into a single-cell suspension according to the digestion method presented in Potts et al.^66^^,^^77^. Briefly, ventricles were digested using a buffer containing PBS, taurine, glucose, 2,3-butanedione monoxime, HEPES, CaCl₂, collagenase II/IV and DNase I. Tissue was digested at 32 °C, and dissociated cells were filtered using 100-μm filters before resuspension in DMEM with 10% foetal bovine serum. Three hearts were pooled per fish per timepoint. Filter size was selected after systematic optimisation to retain cardiomyocytes. Cell viability was assessed prior to loading. The number of cells and quality metrics are provided in Supplementary Fig. 6.

Single-cell RNA sequencing was performed using the 10X Chromium Next GEM Single Cell 3′ v3.1 kit. Sequencing was conducted on the Illumina NextSeq 500/550, achieving ~50,000 reads per cell. FASTQ files were generated using Cell Ranger mkfastq (v3.0.2), and reads were aligned to updated *A. mexicanus* genome assembly (AstMex3) with mitogenome appendance and custom 3′UTR-extended annotations^77^

Analysis was conducted using Seurat (v4.0.6)^78^. Quality control removed low-quality cells based on average nFeatures and nCounts, with a mitochondrial content threshold of <%20 for cardiomyocytes and <%10 for leukocytes. Ambient RNA was removed using decontX, and doublets were identified and removed using scDblFinder prior to downstream analysis. Data were normalised using SCTransform with ribosomal genes regressed out^79^. Batch effects were corrected for during integration of all 12 samples using Harmony (v1.0^69^ to create a final data of 60,598 cells. The integrated dataset was centred and scaled prior to dimensional reduction using PCA and UMAP. Appropriate cell cluster resolution was set using Clustree^80^, and cell clusters were annotated based on canonical marker genes established in zebrafish and validated previous *A. mexicanus* studies. Differential proportion analysis was performed according to the methodology presented in Farbehi et al.^81^. Differential gene expression was analysed using MAST^82^. Gene ontology and^73^ and pathway analysis were performed using fGSEA (v1.16.0)^74^, and PROGENy (v1.12.0)^31^.

### Drug administration

Water-soluble Dexamethasone was administered at a concentration of 10 mg/L in system water for 7 consecutive days. The Dexamethasone solution and control system water were replenished daily for both the treatment and control groups.

Clodronate liposomes (5 mg Clodronate/mL) and Dil-fluorophore tagged control PBS liposomes (Liposoma, the Netherlands) were administered via intraperitoneal injection at 10 µL per fish between 7- and 14-dpci. Dil fluorescent PBS liposomes were injected from 7- to 11 dpci for 5 consecutive days to visualise affected cell populations. In a separate cohort, Clodronate and Dil-tagged PBS liposomes were mixed at 1:1 ratio and co-injected at 10 µL total per fish between 7- and 14-dpci to assess liposome uptake by targeted cell populations. Hearts were isolated at 14- and 30-dpci for RNAscope and immunostaining.

2-acetamidophenol (AMP) was administered via intraperitoneal injection at 10 µL per fish between 7- and 14-dpci and co-injected with Dil-tagged PBS liposomes to assess phagocytic activity in vivo. AMP was dissolved in saline at a concentration of 50 mg/kg, with injection volumes adjusted per fish body weight. Control fish received saline and Dil liposomes at the same volume. Hearts were isolated 14-dpci and 30-dpci for analysis.

Acalabrutinib was administered intraperitoneally at a concentration of 5 mg/kg between 7- and 11-dpci for the main experiment, with additional cohorts treated from 2- to 5-dpci and 26- to 30-dpci to define the critical window of B cell activity. Hearts were isolated at 5-dpci, 14-dpci and 30-dpci as appropriate. A working solution was prepared by diluting a 10 mg stock solution (1:10 DMSO:corn oil) and further diluting in corn oil, with the concentration adjusted per fish body weight. The injection solution was sonicated in a Bioruptor Pico ultrasonicator using the following program: 30 s of sonication at ultrahigh frequency, followed by a 30-s break, repeated for 30 cycles at 20 °C. Fresh injection solutions were prepared daily and sonicated for at least 3 cycles in the provided program to prevent clogging. Control fish received the equivalent volume of DMSO:corn oil vehicle.

DHMEQ was injected intraperitoneally at 10 mg/kg in a 10 µL per fish, daily between 7- and 14-dpci for the 14-dpci time point, and between 7- and 30-dpci for the 30-dpci time point. The concentration was determined as 10 mg/kg, with a working solution of 0.8 mg/L. A stock solution was prepared by dissolving DHMEQ in DMSO:PEG300:Tween80:saline (0.1:0.3:0.05:0.455) and further diluted in saline, with the concentration adjusted per fish body weight. Control fish received the equivalent volume of solvent diluted in saline buffer daily.

### Tissue processing

After isolation, hearts were briefly washed in PBS and fixed overnight in 4% paraformaldehyde at 4 °C. The following day, samples were rinsed three times in PBS for 5 min each and dehydrated in an ascending ethanol series (70, 80, 90, 96 and 100%) and 100% butanol for 1 h per step, followed by overnight storage in 100% butanol at 4 °C.

Samples were then transferred to paraffin wax (Paraplast Plus, Sigma-Aldrich P-3683) at 65 °C. The paraffin was renewed three times at 1-h intervals, and hearts were embedded in sectioning moulds. After the wax solidified, sections were cut to 7 µm thickness using a Leica microtome and stored in stackable trays.

For Dil fluorophore analysis, hearts were briefly washed in PBS and fixed in 4% PFA for 2 h at room temperature. The following day, hearts were rinsed in 5% sucrose for 30 min and incubated in 30% sucrose overnight. The following day, samples were embedded in OCT, and cryosections were cut at 7 µm thickness and stored at −80 °C.

### Histology

Acid Fuchsin Orange-G (AFOG) staining was performed as previously described by Poss et al.^1^. Hearts were mounted as 1 in 10 sections on Superfrost glass slides and left on a hotplate overnight. Samples were deparaffinised in xylene twice for 5 min each, followed by a descending ethanol series (100, 96, 90, 80 and 70%) for 1 min each. Slides were placed in MilliQ water and refixed in Bouin’s solution for 2 h at 60 °C. After cooling, slides were washed in MilliQ water for 30 min, incubated in 1% phosphomolybdic acid for 5 min, rinsed with MilliQ water, stained with AFOG solution for 9 min, and briefly dehydrated in an ascending ethanol series. AFOG solution was prepared by boiling 1 L of MilliQ water with 5 g of methyl blue, followed by the addition of 10 g of Orange-G and 15 g of Acid Fuchsin after cooling. The pH was adjusted to 1.09 using HCl. Following Histoclear treatment for 10 min, sections were mounted in DPX mounting medium and left to dry overnight.

### Immunofluorescent staining

For immunofluorescent staining, multiple sections were mounted on slides and left on a hotplate at 37 °C overnight. Slides were deparaffinised in two xylene washes (5 min each) and rehydrated in a descending ethanol series. Samples were immersed in MilliQ water and pressure-cooked for 8 min in Antigen Unmasking Solution (Vector, H-3300 citrate-based, pH 6). Slides were cooled and transferred to 0.01% PBS-Tween80 for 5 min. Sections were circled with a pap-pen and blocked in TNB buffer (0.1 M Tris-HCl, pH 7.5, 0.15 M NaCl, 0.5% blocking reagent; Perkin Elmer) for at least 30 min at room temperature. Primary antibodies were diluted 1:200 in TNB and applied to sections, which were incubated overnight at room temperature in a humidified box. Slides were washed three times in 0.01% PBS-Tween 80 for 5 min each, followed by incubation with secondary antibodies (1:250 in TNB) for 2 h. Sections were counterstained with DAPI, washed three times in 0.01% PBS-Tween 80, and mounted in Mowiol for imaging. For wholemount imaging of IgM^+^ cells, hearts from Tg(*Cau.ighv-ighm:EGFP*)^42^ fish were dissected into ice-cold PBS, fixed in 4% Paraformaldehyde (PFA) at 4 °C, then further washed in PBS prior to immunostaining. Primary antibodies used included MOUSE ANTITI-mf20 (1:200, DSHV, AB_2147781), mouse anti-PCNA (1:200, Dako, M0879), rabbit anti-Mef2 (1:200, Santa Cruz, SC-313), rabbit anti-transgelin (1:200, Abcam, ab14106), and chicken anti-GFP (1:100, Abcam, ab13970). Secondary antibodies were goat anti-mouse Alexa Fluor 488 (1:250, Invitrogen, A11001/A55058), donkey anti-rabbit Alexa Fluor 546 (1:250, Invitrogen A10040), goat anti-mouse Alexa Fluor 555 (1:250, Invitrogen, a31570), and goat anti-chicken 488 (1:500, Thermo, A11039).

### RNAscope in situ hybridisation

RNAscope (Advanced Cell Diagnostics, Hayward, CA) was used for in situ hybridisation on paraffin-embedded sections (7 µm thick). Multiple sections were mounted per sample and deparaffinised in two xylene washes (5 min each), followed by two 2-min washes in 100% ethanol. After air-drying, sections were treated with hydrogen peroxide for 10 min at room temperature and boiled in RNAscope Target Retrieval Solution for 15–18 min at 96–102 °C. Slides were washed in 100% ethanol and incubated with RNAscope Protease III for 15 min at 40 °C. Sections were then incubated with the probe mix for 2 h and treated with amplification buffers and hybridisation reagents at 40 °C according to the manufacturer’s instructions (RNAscope Multiplex Detection Kit). Hybridisation signals were detected using RNAscope Multiplex Fluorescent Detection Reagents v2 with TSA Plus Cyanine 3, Cyanine 5, and Fluorescein fluorophores (Perkin Elmer, NEL744001KT). All *A. mexicanus* custom probes were designed by Advanced Cell Diagnostics (Biotechne). Samples were counterstained with myocardial marker MF-20 (myosin heavy chain, 1:200, HSHB AB-2147781) and DAPI, then mounted using gold anti-fade mountant.

### Image acquisition and analysis

AFOG images were acquired using a Nikon DS-Fi3 Light Microscope (4× and 10× objectives). Regeneration capacity was assessed by dividing the total wound area by the total ventricle area, expressed as a percentage or a ratio.

Confocal microscopy was performed using a Zeiss 880. Zebrafish imaging was performed on a Leica TCS SP8 AOBS confocal laser scanning microscope using a 10×/0.4 HC PL APO Dry or 20×/0.75 HC PL APO CS2 Immersion objective. Imaging of AFOG staining on sections was performed on an Evident (Olympus) VS200 slide scanner microscope with colour camera and 20×/0.8 dry objective.

Fiji/ImageJ software was used for image processing, blinding, wound and ventricular area measurements and manual quantifications. MF20 was used as a reference to define the wound area. Nuclei colocalised with positive signals were manually quantified using the multipoint tool in Fiji. Counts were normalised to fractional wound area (wound area/total ventricle area). Values from different sections of the same heart were averaged.

### Statistical tests and directionality

Sample sizes are reported as discrete numbers in figure legends and Supplementary Table 2 throughout the manuscript. For histological quantifications, *n* refers to individual hearts (biological replicates). For scRNA-seq, sample numbers are detailed in Supplementary Fig. 6. All *n* values represent distinct biological samples. All measurements were taken from distinct biological samples. Each heart was used for a single time point and condition. No repeated measures were taken from the same animal. Two-tailed tests were used throughout unless otherwise stated. Tests used include: two-tailed Mann–Whitney *U* tests for non-normally distributed data, two-tailed unpaired Student’s *t* tests (with Welch’s correction where indicated) for normally distributed data, and two-way ANOVA with Tukey’s post-hoc correction for multiple comparisons. Two-sided permutation-based differential proportion analysis was performed as described in Farbehi et al.^71^. For each timepoint, the observed difference in the proportion of each cell population between surface fish and Pachón was compared against a null distribution generated by 1000 random permutations of cell-type labels, *p*-values were corrected for multiple comparisons across the six cell populations within each timepoint using the Benjamini–Hochberg procedure and are reported as FDR. Effect sizes are reported as the log2 fold difference in proportion. Fold changes relative to uninjured within each morph are descriptive ratios of estimated proportions and were not subjected to hypothesis testing. Because three hearts were pooled per morph per timepoint prior to library preparation, DPA compares cell proportions within pooled libraries and the unit of analysis is the cell rather than the individual animal; independent per-animal quantification of leukocyte populations is provided by RNAscope in Figs. 1f and 2g, i, k.

Pearson’s correlation was used for the B cell score vs. G1-to-S phase analysis (Fig. 5j). All tests are described by name in figure legends; the differential proportion analysis and deconvolution methodology are described in detail in the 'Methods'. No covariates were included in the primary statistical comparisons. For the scRNA-seq analysis, batch effects between samples were corrected using Harmony prior to downstream analysis. Ribosomal gene expression was regressed out during SCTransform normalisation. Parametric tests (Student’s *t* test) were applied where data were normally distributed; Mann–Whitney *U* tests were used for non-normal distributions. For multiple comparisons in two-way ANOVA, Tukey’s post-hoc correction was applied. For differential proportion analysis, FDR correction was applied using permutation testing with 95% bootstrap confidence intervals. GraphPad Prism (version 10.1.1, Boston, Massachusetts, USA, www.graphpad.com) was used for visualisation.

### Bisque deconvolution and analysis

For single-cell, FASTQ files were mapped to the *Astyanax mexicanus* genome (AstMex3) with the mitogenome (after removing rRNA and tRNA regions) appended. A custom Snakemake pipeline managed the workflow using cell-ranger (v8.0.1) for alignment and quantification. Bulk RNA-seq samples were similarly processed using FASTQC and Trim-Galore for quality control and trimming, then mapped with STAR (v2.7.11b) and normalised with DESeq2. Both scRNA-seq and bulk RNA-seq data were mapped to the genome version (AstMex3) to ensure consistency between analyses.

In single cell preprocessing, ambient RNA was removed with decontX and doublets identified via scDblFinder. Quality control thresholds were applied (mitochondrial content <25% for cardiomyocytes and nCount/nFeature within ±2 standard deviations), followed by normalisation using SCTransform (regressing out ribosomal genes) and integration with Harmony (Supplementary Fig. 6). Cell-type annotation was performed using Seurat’s FindAllMarkers, with cxcr4b expression defining broad leukocyte populations and a stricter mitochondrial threshold (<10%) applied to leukocytes; these annotations were then merged into a master reference.

For bulk deconvolution, 100 markers per cell population were identified from the master single-cell dataset using Seurat’s FindAllMarkers. Bisque was then applied—requiring at least 25 unique markers per population—to estimate cell-type proportions in each bulk RNA-seq sample. A correlation and pathway analysis were conducted by performing Spearman’s rank correlation between B cell deconvolution scores and all genes, highlighting those with coefficients >0.7. The ranked gene list was used in fGSEA with WikiPathways to identify the top 20 enriched pathways B cell scores were additionally correlated with GSVA summary scores for the G1 to S Cell Cycle Control pathway, with Pearson’s correlation coefficient and *p*-value calculated.

### Reporting summary

Further information on research design is available in the Nature Portfolio Reporting Summary linked to this article.

## Supplementary information

Supplementary Information

Peer Review File

Reporting Summary

## Source data

Source Data

## Data Availability

The raw single-cell RNA-sequencing data generated in this study have been deposited in the Gene Expression Omnibus (GEO) database under accession code GSE291684 [https://www.ncbi.nlm.nih.gov/geo/query/acc.cgi?acc=GSE291 684]. All data and material requests should be directed to Mathilda Mommersteeg (mathilda.mommersteeg@dpag.ox.ac.uk). The Tg(*Cau.ighv-ighm:EGFP*)^42^ and *rag*^*hu1999*^^43^ mutants are available from Rebecca Richardson (rebecca.richardson@bristol.ac.uk). Source data are provided with this paper.
