# Supplementary material for: Absence of a prolonged macrophage and B cell response inhibits heart regeneration in the Mexican cavefish
Source: Nat Commun. 2026 Sep 17;17:10922. doi: 10.1038/s41467-026-77725-4 (PMC13680611; doi:10.1038/s41467-026-77725-4)
Supplement: Supplementary file 1 [file 41467_2026_77725_MOESM1_ESM.pdf]

Supplementary Information

Absence of a prolonged macrophage and B cell response inhibits heart regeneration in *the Mexican cavefish*

Sengul et al.

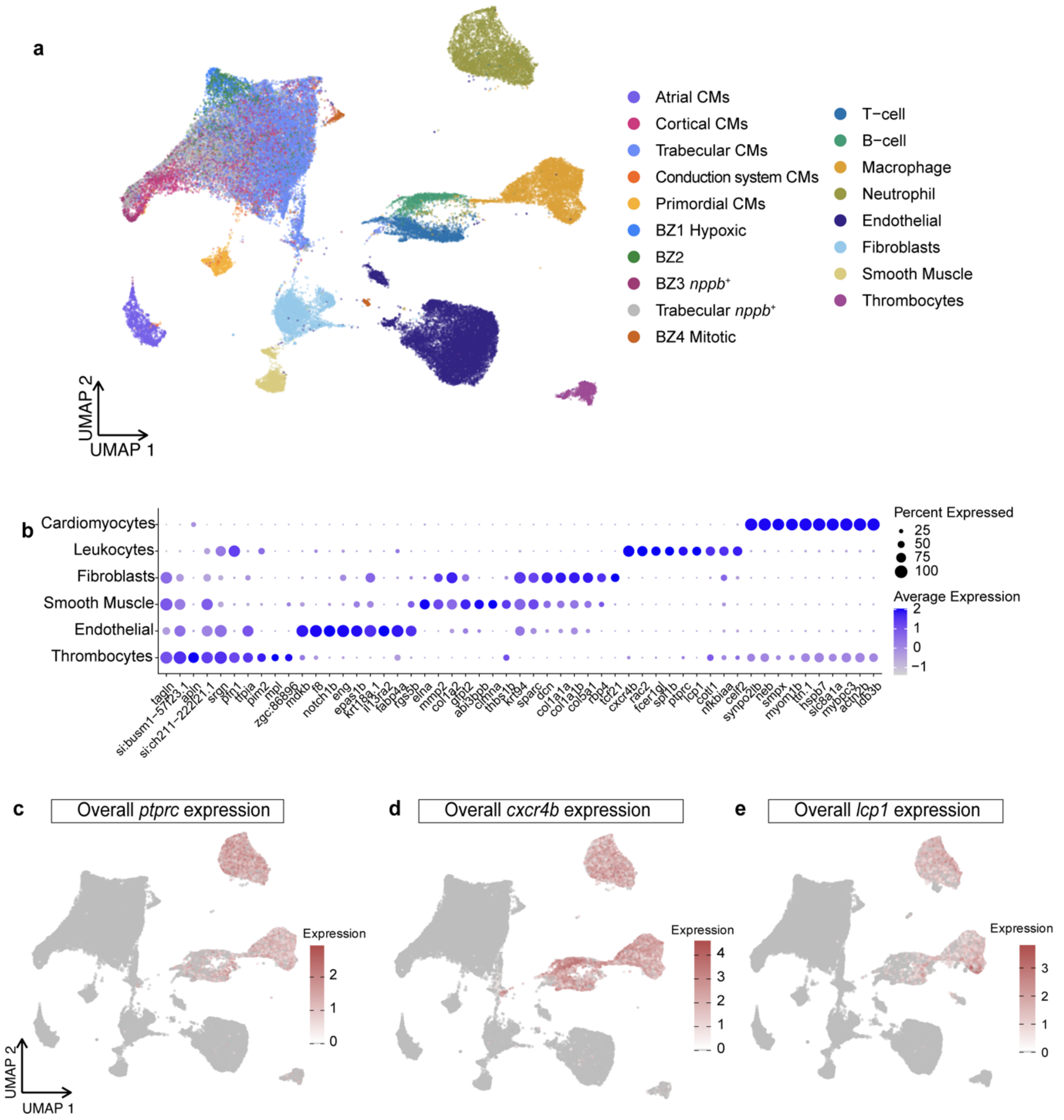

Supplementary Fig. 1 | Single cell profiling of *Astyanax mexicanus* heart cell populations. **a** UMAP visualisation of all annotated cell populations in the *A. mexicanus* heart. **b** Unbiased dot plot displaying the expression of top defining marker genes for each cell type annotated in Figure 1b. **c-e** UMAP plot highlighting the expression of *ptprc* (c), *cxcr4b* (d), and *lcp1* (e) across all cell populations in the heart.

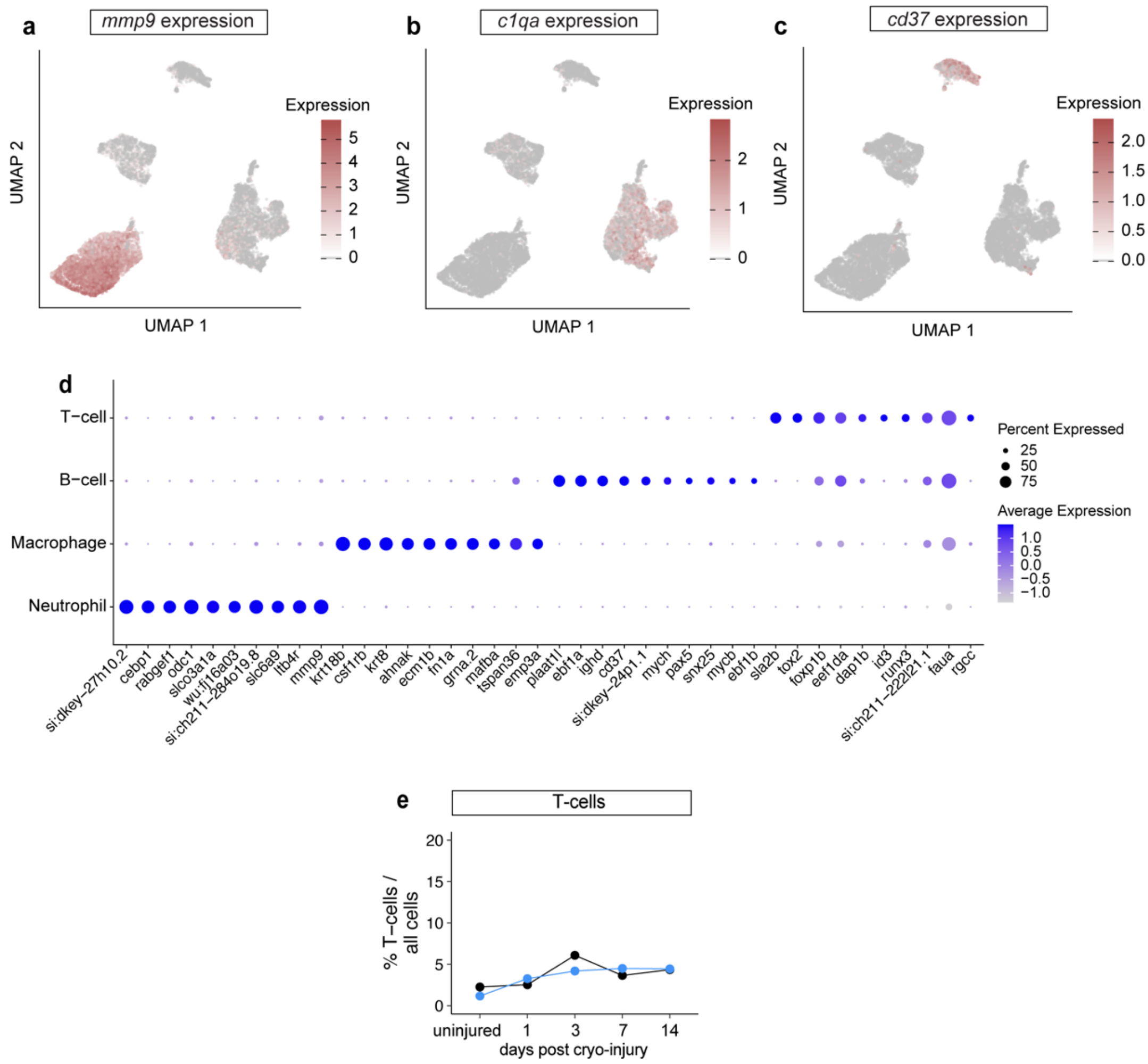

Supplementary Fig. 2 | Leukocyte sub-population characterisation in *Astyanax mexicanus* hearts. **a-c** UMAPs showing the overall expression of *mmp9* (a), *c1qa* (b), and *cd37* (c) across all leukocyte sub-populations, confirming these markers for neutrophils, macrophages and B cells, respectively. **d** Dot plot displaying the expression of canonical marker genes used to define each leukocyte population. Dot size represents the percentage of cells expressing each gene; color intensity represents average scaled expression. Marker genes are shown for neutrophils, macrophages, B cells, and T cells. **e** Differential proportion analysis of T cells as a percentage of total cells at uninjured, 1-, 3-, 7-, and 14-dpci in Pachón and surface fish, showing significant differences between morphs across the time course.

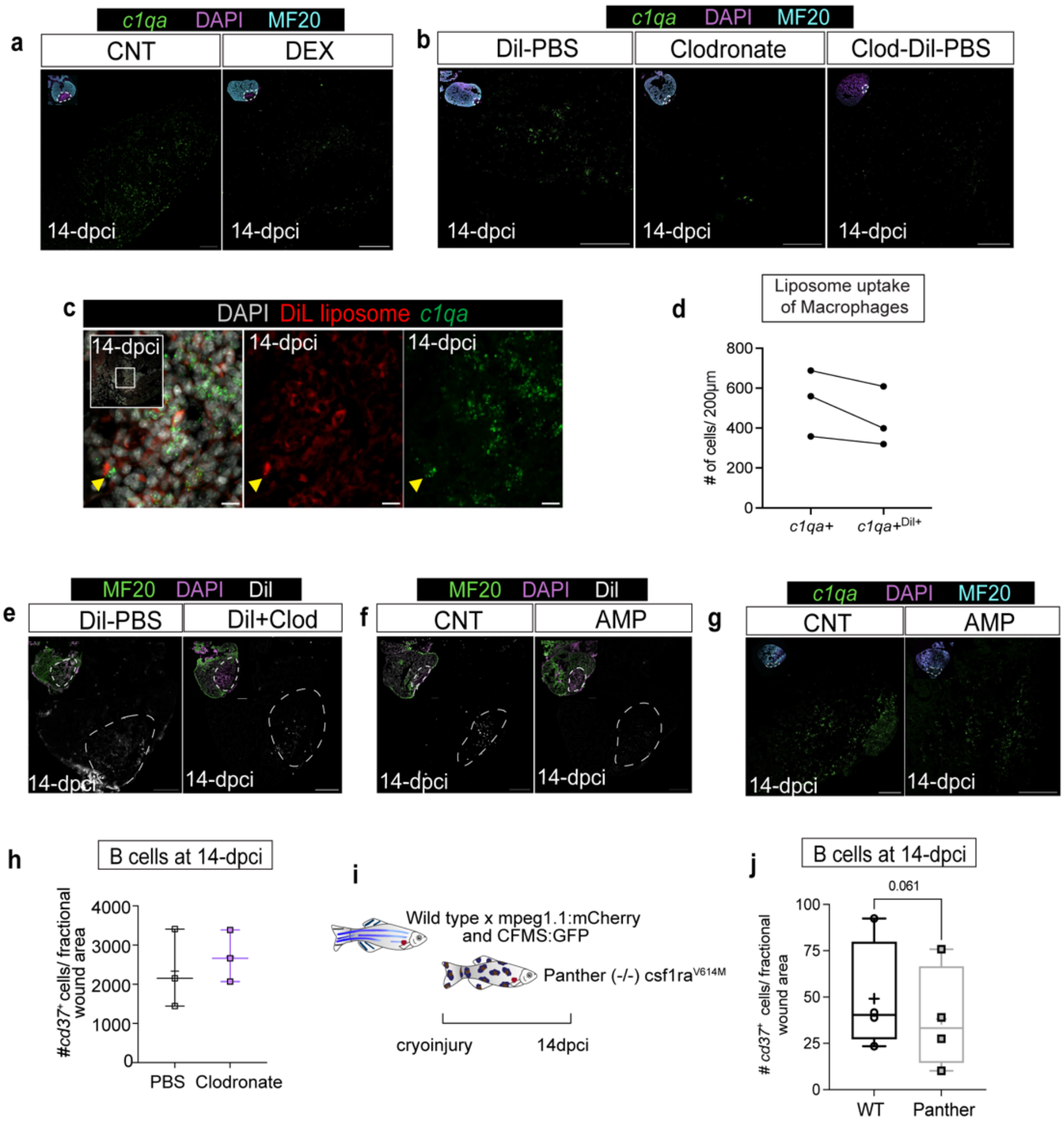

Supplementary Fig. 3 | Supporting data for the macrophage phagocytosis experiments. **a** RNAscope images showing *c1qa* macrophages (green) in control and Dexamethasone-treated surface fish hearts at 14-dpci, confirming no significant difference in macrophage numbers upon Dexamethasone treatment. Scale bar, 100 µm. **b** RNAscope images showing *c1qa*+ macrophages (green) in Dil-PBS, Clodronate, and Clodronate+Dil-PBS treated hearts at 14-dpci, confirming macrophage depletion by Clodronate treatment. Scale bar, 100 µm. **c** Confocal images showing co-localisation of Dil liposomes (red) and *c1qa*+ macrophages (green) with DAPI (grey) at 14-dpci. Yellow arrowhead indicates a macrophage that has ingested Dil liposomes. Scale bar, 20 µm. **d** Quantification of total *c1qa*+ cells compared to *c1qa*+Dil+ double-positive cells, confirming the majority of late-stage macrophages are actively phagocytic; 75% of macrophages colocalised with DiL liposome, confidence Interval: 58.80% to 90.35% (n=3). **e-f** Representative whole-heart images showing Dil liposome signal with MF20 (green) to represent the wound region and DAPI (magenta) in Dil-PBS and Clodronate+Dil-PBS (**e**) and Control and AMP (**f**) confirms that liposome uptake across the wound is dependent on the presence of phagocytic macrophages and phagocytosis, respectively. Dashed line indicates the wound region Scale bar, 100 µm. **g** Representative RNAscope images showing *c1qa*+ macrophages (green) in control and AMP-treated hearts at 14-dpci, confirming that macrophage numbers are not affected by AMP treatment. Scale bar, 100 µm. **h** Quantification of *cd37*+ B cells in PBS and Clodronate-treated hearts at 14-dpci, showing no significant difference. **i** Experimental schematic showing cryoinjury timeline for wild-type and Panther (*csf1ra*-/-) zebrafish with heart collection at 14-dpci. **j** Quantification of *cd37*+ cells showing no difference in B cell response between the control and Panther zebrafish. Source data for panels d, h, j are provided as a Source Data file.AMP, 2-acetamidophenol; CNT, control; dpci, days post-cryoinjury; fwa, fractional wound area.

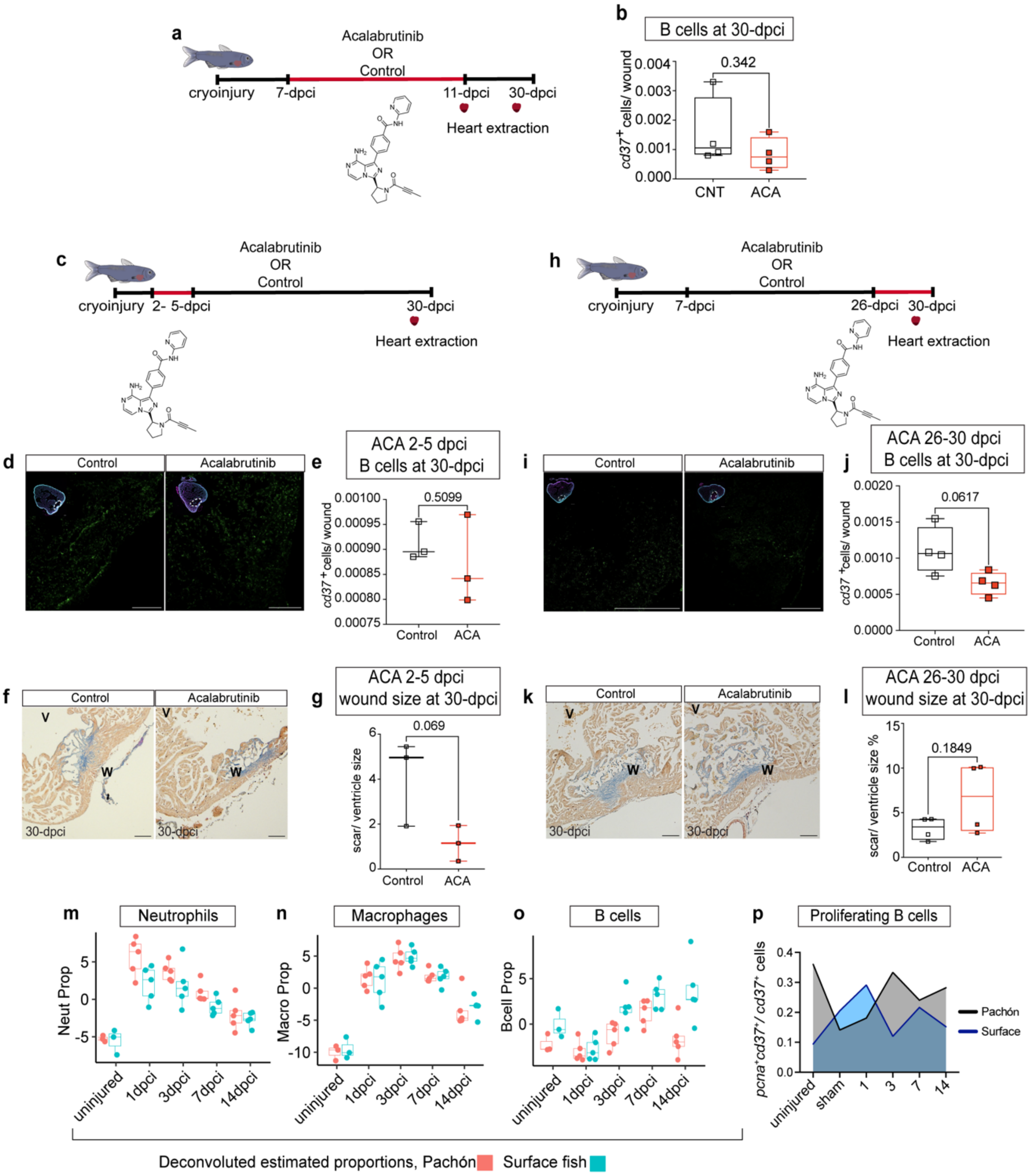

Supplementary Fig. 4 | Additional Acalabrutinib experiments and deconvolution analysis.
a Experimental schematic: surface fish were injected with Acalabrutinib or control from 7- to 14-dpci, with hearts collected at 30-dpci. **b** Quantification of *cd37*⁺ B cells normalised to the wound area at 30-dpci in Control and Acalabrutinib-injected surface fish hearts, showing no significant difference at this timepoint. Student's t-test, p=0.342; n=4 per group. **c** Experimental schematic: surface fish were injected with Acalabrutinib or control from 2- to 5-dpci prior to B cell recruitment, with hearts collected at 30-dpci. **d** Representative RNAscope images showing *cd37*⁺B cells (green) in control and Acalabrutinib treated hearts from the 2-5-dpci treatment group. Scale bar, 100 μm. **e** Quantification of *cd37*⁺ B cells normalised to the wound area at 30-dpci in the 2-5-dpci treatment groups, showing no significant difference. Student's t-test, p=0.510; n=3 per group. **f** AFOG-stained sections of control and Acalabrutinib-treated hearts from 2-5dpci treatment group at 30-dpci. Scale bar, 100 μm. **g** Quantification of wound area at 30-dpci in the 2-5dpci Acalabrutinib treatment group, showing no significant difference in wound size. Student's t-test, p=0.069; n=3 per group. **h** Experimental schematic: surface fish were injected with Acalabrutinib or control from 26- to 30-dpci, during late B cell activity, with hearts collected at 30-dpci. **i** Representative RNAscope images showing *cd37*⁺ B cells (green) in control and Acalabrutinib treated hearts from the 26-30-dpci treatment group. Scale bar, 100 μm. **j**

Quantification of *cd37*$^+$ B cells normalised to the wound area at 30-dpci in the 26-30-dpci treatment groups, showing no significant difference. Student's t-test, p=0.062; n=4 per group. **k** AFOG-stained sections of control and Acalabrutinib-treated hearts from 26-30dpci treatment group at 30-dpci. Scale bar, 100 µm. **l** Quantification of wound area at 30-dpci in the 26-30dpci Acalabrutinib treatment group, showing no significant difference in wound size. Student's t-test, p=0.1527; n=4 per group. **m-o** Deconvoluted differential proportion analysis showing the relative abundance of neutrophils (**m**), macrophages (**n**), and B cells (**o**) at uninjured, 1-, 3-, 7-, and 14-dpci in surface and Pachón, confirming the trends observed in the single-cell data. **p** Proportion of proliferating B cells (*pcna*$^+$*cd37*$^+$) to total B cells (*cd37*$^+$) in surface fish and Pachón ventricles across the time course, derived from scRNA-seq data, showing that B cells themselves do not have differences in proliferation between two morphs during the regenerative response.

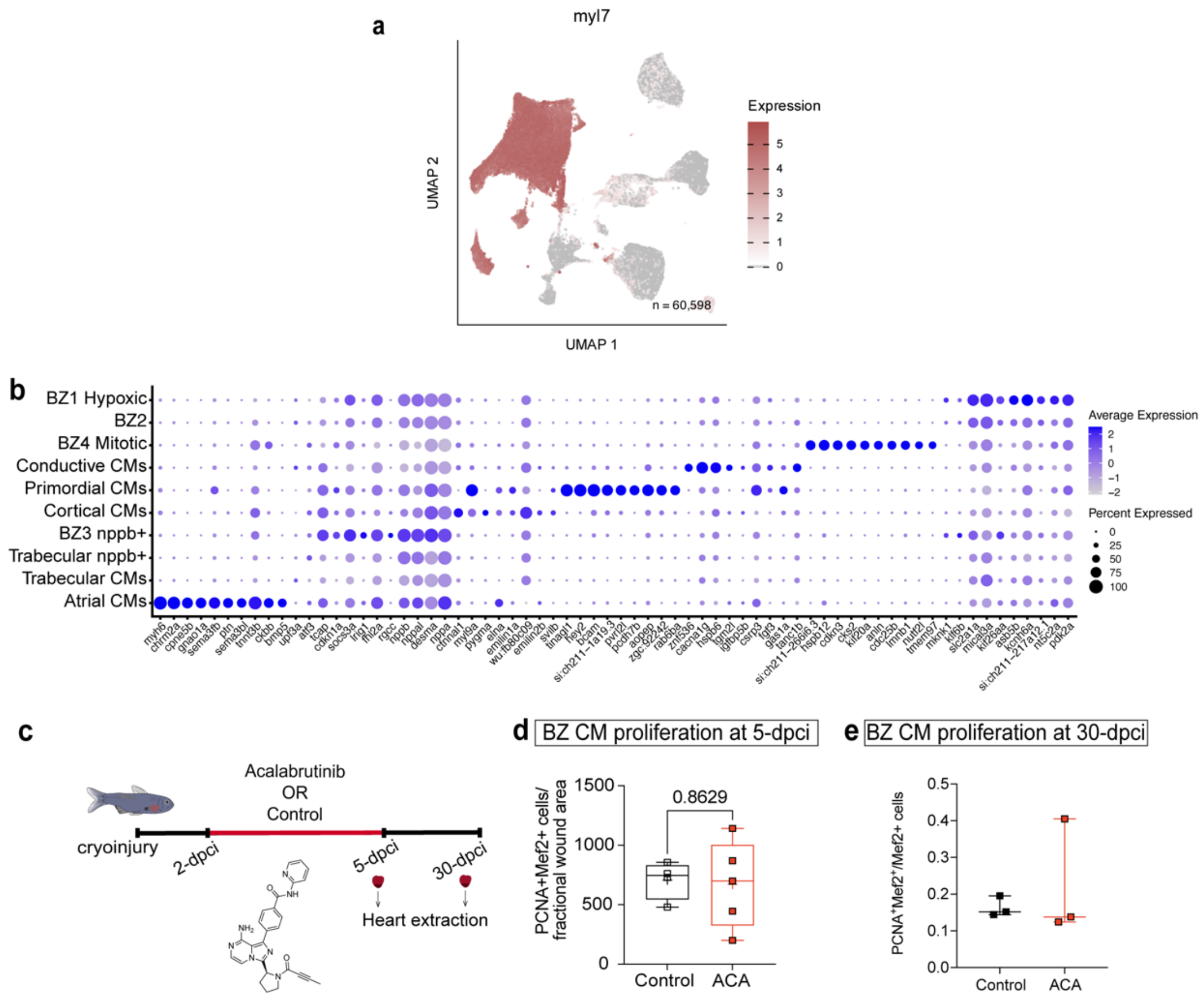

Supplementary Fig. 5 | Cardiomyocyte sub-population characterisation and Acalabrutinib off-target controls. **a** UMAP of re-clustered cardiomyocyte scRNA-seq data showing widespread expression of *myl7*, confirming successful isolation of cardiomyocytes. **b** Dot plot displaying the expression of canonical marker genes used to define each cardiomyocyte sub-population. Dot size represents the percentage of cells expressing each gene; colour intensity represents average scaled expression. **c** Experimental schematic: surface fish were treated with Acalabrutinib or control from 2- to 5-dpci, prior to B cell recruitment. Hearts were collected at 5-dpci and 30-dpci for immunostaining. **d** Quantification of $PCNA^{+}Mef2^{+}$ border zone cardiomyocytes at 5-dpci normalised to fractional wound area at 5-dpci in control and Acalabrutinib-treated hearts, showing no significant difference. Unpaired t-test, p=0.863; n=4-5 per group. **e** Quantification of $PCNA^{+}Mef2^{+}$ border zone cardiomyocytes at 30-dpci in control and Acalabrutinib-treated hearts from the 2-5-dpci treatment group, showing no significant difference, confirming the absence of off-target effects of Acalabrutinib on cardiomyocyte proliferation. Unpaired t-test, p=0.5613; n=3 per group.

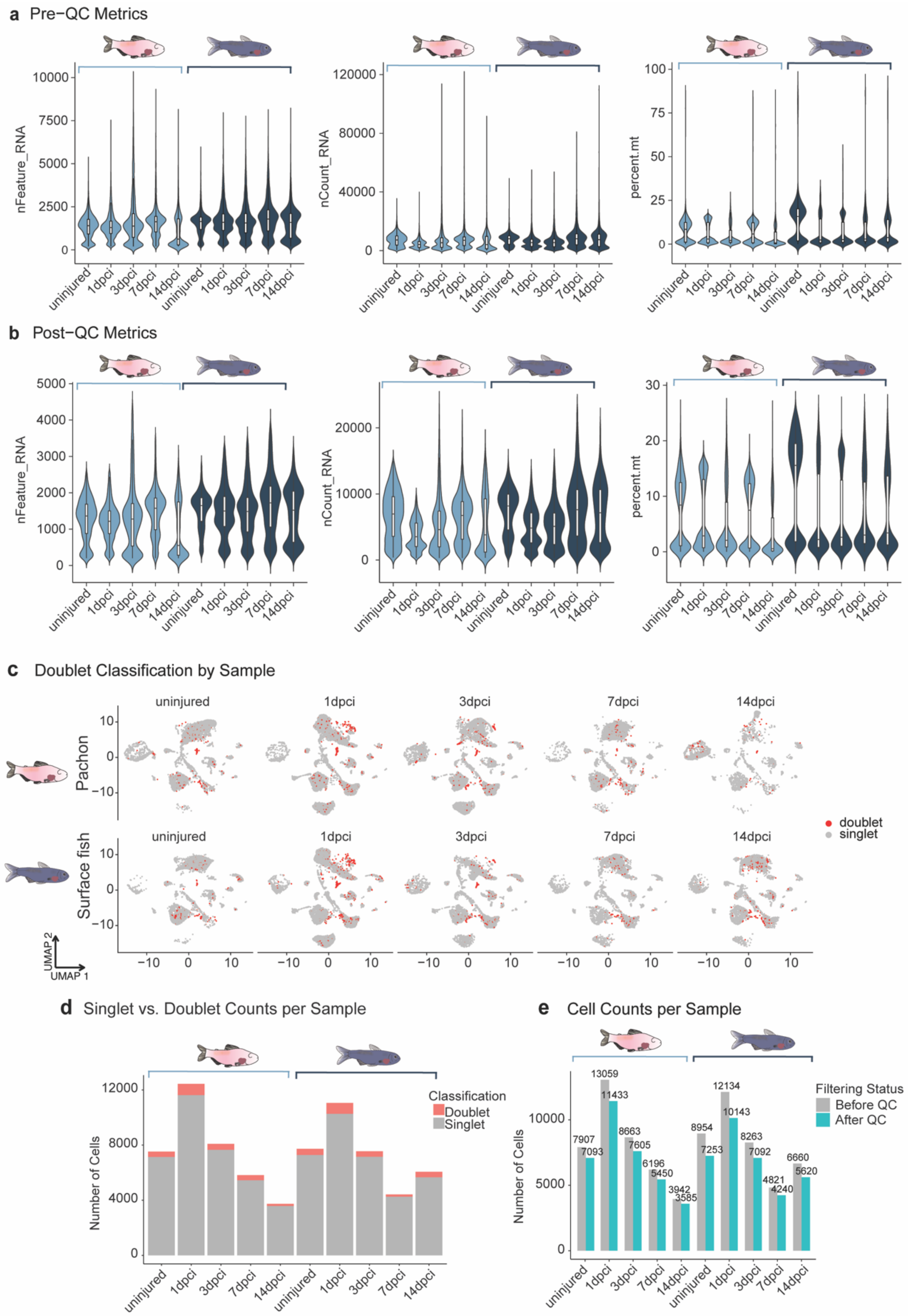

Supplementary Fig. 6 | scRNA-seq quality control metrics for all samples. a Violin plots showing distribution of quality control metrics before filtering across samples of Pachón and surface fish at all time points, including the number of RNA molecules per cell (nCount_RNA), number of genes detected per cell (nFeature_RNA), and percentage of mitochondrial reads. B Violin plots showing same three quality control metrics after filtering, nCount_RNA (left, nFeature_RNA (center), and mitochondrial percentage (right), demonstrating successful removal of low-quality cells, empty droplets, and doublets. Thresholds applied nCount_RNA and nFeature_RNA within ±2 standard deviations of the sample mean;

mitochondrial content <20% for cardiomyocytes and <10% for leukocytes. C UMAPs showing doublet classification from scDblFinder across all samples in Pachón (top row) and surface fish (bottom row) at uninjured, 1-, 3-, 7-, and 14-dpci. Predicted doublets (red) are distributed across cell populations without clustering in specific regions, confirming that doublet removal did not disproportionally affect any particular cell type. D Bar charts showing total cell numbers per sample before (grey) and after (red) quality control filtering across Pachón and surface fish samples, illustrating the proportion of cells removed at each step. E Bar charts showing the number of cells per sample before (grey) and after (blue) quality control with detected number of genes per sample, confirming the consistent sequencing depth and cell recovery across all samples.

Article title: Absence of a prolonged macrophage and B cell response inhibits heart regeneration in *the Mexican cavefish*

Sengul et al.

Supplementary table 1| Overview of permutation values displayed on the DPA figures.

**Figure**	**Analysis**	**Comparisons**	**Time point**	**FDR/ fold change**
**Fig. 1d**	DPA leukocytes	Surface vs Pachón	7 dpci	0.0015
**Fig. 1d**	DPA leukocytes	Pachón	uninjured vs 1-dpci	4.30×
**Fig. 1d**	DPA leukocytes	Pachón	uninjured vs 3-dpci	5.69×
**Fig. 1d**	DPA leukocytes	Surface fish	uninjured vs 1-dpci	7.73×
**Fig. 1d**	DPA leukocytes	Surface fish	uninjured vs 3-dpci	6.80×
**Fig. 1d**	DPA leukocytes	Surface fish	uninjured vs 7-dpci	5.50×
**Fig. 1d**	DPA leukocytes	Surface fish	uninjured vs 14-dpci	4.59×
**Fig. 2c**	DPA neutrophils	Surface vs Pachón	3-dpci	0.0018
**Fig. 2c**	DPA neutrophils	Surface vs Pachón	7-dpci	0.0015
**Fig. 2c**	DPA neutrophils	Surface vs Pachón	14-dpci	0.003
**Fig. 2d**	DPA macrophages	Surface vs Pachón	7-dpci	0.0015

Supplementary table 2| Overview of statistical tests and values displayed on the figures.

Figure	Analysis	Comparison & distinct sample size	Time point	p-value	Test Type
**Fig. 1f**	*ptprc*$^{+}$ cells	Surface fish (n=3) vs Pachón (n=3)	1-dpci	0.2	Two-sided Mann-Whitney U
**Fig. 1f**	*ptprc*$^{+}$ cells	Surface fish (n= 5) vs Pachón (n= 4)	3-dpci	0.7302	Two-sided Mann-Whitney U
**Fig. 1f**	*ptprc*$^{+}$ cells	Surface fish (n= 5) vs Pachón (n= 6)	7-dpci	0.1775	Two-sided Mann-Whitney U
**Fig. 1f**	*ptprc*$^{+}$ cells	Surface fish (n= 8) vs Pachón (n= 5)	14-dpci	0.0016	Two-sided Mann-Whitney U
**Fig. 1f**	*ptprc*$^{+}$ cells	Surface fish (n=7) vs Pachón (n=5)	30-dpci	0.048	Two-sided Mann-Whitney U
**Fig. 1f**	*ptprc*$^{+}$ cells	Surface fish (n=3) vs Pachón (n= 5)	60-dpci	0.0357	Two-sided Mann-Whitney U
**Fig. 1f**	*ptprc*$^{+}$ cells	Pachón (n=3) vs uninjured (n=3)	1-dpci	0.083	Two-sided Mann-Whitney U
**Fig. 1f**	*ptprc*$^{+}$ cells	Pachón (n=4) vs uninjured (n=3)	3-dpci	0.0571	Two-sided Mann-Whitney U
**Fig. 1f**	*ptprc*$^{+}$ cells	Pachón (n=6) vs uninjured (n=3)	7-dpci	0.0238	Two-sided Mann-Whitney U
**Fig. 1f**	*ptprc*$^{+}$ cells	Pachón (n=5) vs uninjured (n=3)	14-dpci	0.0357	Two-sided Mann-Whitney U
**Fig. 1f**	*ptprc*$^{+}$ cells	Pachón (n=5) vs uninjured (n=3)	30-dpci	0.0357	Two-sided Mann-Whitney U
**Fig. 1f**	*ptprc*$^{+}$ cells	Pachón (n=5) vs uninjured (n=3)	60-dpci	0.0357	Two-sided Mann-Whitney U
**Fig. 1f**	*ptprc*$^{+}$ cells	Surface fish (n=3) vs uninjured (n=3)	1-dpci	0.1	Two-sided Mann-Whitney U
**Fig. 1f**	*ptprc*$^{+}$ cells	Surface fish (n=5) vs uninjured (n=3)	3-dpci	0.0357	Two-sided Mann-Whitney U
**Fig. 1f**	*ptprc*$^{+}$ cells	Surface fish (n=5) vs uninjured (n=3)	7-dpci	0.0357	Two-sided Mann-Whitney U

**Fig. 1f**	*ptprc*$^+$ cells	Surface fish (n=8) vs uninjured (n=3)	14-dpci	0.0121	Two-sided Mann-Whitney U
**Fig. 1f**	*ptprc*$^+$ cells	Surface fish (n=7) vs uninjured (n=3)	30-dpci	0.0167	Two-sided Mann-Whitney U
**Fig. 1f**	*ptprc*$^+$ cells	Surface fish (n=3) vs uninjured (n=3)	60-dpci	0.1	Two-sided Mann-Whitney U
**Fig. 1i**	Wound size	Control (n=5) vs DEX (n=6)	30-dpci	0.044	Two-sided Welsch test
**Fig. 2g**	*mmp9*$^+$ cells	Surface fish (n=6) vs Pachón (n=7)	3-dpci	0.051	Two-sided Mann-Whitney U
**Fig. 2i**	*c1qa*$^+$ cells	Surface fish (n=6) vs Pachón (n=4)	14-dpci	0.0095	Two-sided Mann-Whitney U
**Fig. 2i**	*c1qa*$^+$ cells	Surface fish (n=5) vs Pachón (n=3)	30-dpci	0.0357	Two-sided Mann-Whitney U
**Fig. 2k**	*cd37*$^+$ cells	Surface fish (n=9) vs Pachón (n=8)	30-dpci	0.0001	Two-sided Mann-Whitney U
**Fig. 2k**	*cd37*$^+$ cells	Surface fish (n=6) vs Pachón (n=4)	60-dpci	0.0381	Two-sided Mann-Whitney U
**Fig. 3k**	Wound size	CNT (n= 6) vs DHMEQ (n= 7)	30-dpci	0.366	Two-sided Mann-Whitney U
**Fig. 4a**	*c1qa*$^+$ cells	Control vs DEX	14-dpci	0.4206	Two-sided Mann-Whitney U
**Fig. 4d**	*c1qa*$^+$ cells	PBS (n= 7) vs Clodronate (n= 6)	14-dpci	0.0221	Two-sided Mann-Whitney U
**Fig. 4d**	*c1qa*$^+$ cells	PBS (n= 7) vs PBS+Clodronate (n= 8)	14-dpci	0.0003	Two-sided Mann-Whitney U
**Fig. 4f**	DiI uptake	PBS (n=4) vs PBS+Clodronate (n=6)	14-dpci	0.0190	Two-sided Mann-Whitney U
**Fig. 4h**	Epicardial TAGLN	PBS (n=5) vs Clodronate (n=7)	14-dpci	0.0480	Two-sided Mann-Whitney U
**Fig. 4h**	Epicardial TAGLN	PBS (n=5) vs PBS+Clodronate (n=5)	14-dpci	0.0317	Two-sided Mann-Whitney U
**Fig. 4j**	Wound size	PBS (n=5) vs Clodronate (n=4)	30-dpci	0.0317	Two-sided Mann-Whitney U

**Fig. 4l**	*c1qa*+ cells	CNT (n=5) vs AMP (n=5)	14-dpci	0.3095	Two-sided Mann-Whitney U
**Fig. 4n**	Dil uptake	CNT (n=4) vs AMP (n=4)	14-dpci	0.0286	Two-sided Mann-Whitney U
**Fig. 4p**	Epicardial TAGLN	CNT (n=5) vs AMP (n=5)	14-dpci	0.0079	Two-sided Mann-Whitney U
**Fig. 4r**	Wound size	CNT (n=5) vs AMP (n=4)	30-dpci	0.0159	Two-sided Mann-Whitney U
**Fig. 5a**	*cd37*+ cells	Control (n=3) vs DEX (n=3)	14-dpci	0.0063	Two-sided Student's t-test
**Fig. 5b**	*cd37*+ cells	Control (n=6) vs DEX (n=5)	30-dpci	0.0033	Two-sided Student's t-test
**Fig. 5d**	*cd37*+ cells	Control (n=4) vs ACA (n=4)	14-dpci	0.0265	Two-sided Student's t-test
**Fig. 5f**	Wound size	Control (n=8) vs ACA (n=8)	30-dpci	0.0207	Two-sided Mann-Whitney U
**Fig. 5j**	cell cycle correlation	B cell scores vs G1-S phase	All dpci	R=0.9, p=0.00038	Pearson's correlation
**Fig. 6f**	BZ CM proliferation	Surface fish (n=5) vs Pachón (n=4)	3-dpci	1	Post-hoc Welch’s test
**Fig. 6f**	BZ CM proliferation	Surface fish (n=4) vs Pachón (n=4)	7-dpci	0.402	Post-hoc Welch’s test
**Fig. 6f**	BZ CM proliferation	Surface fish (n=4) vs Pachón (n=4)	14-dpci	0.257	Post-hoc Welch’s test
**Fig. 6f**	BZ CM proliferation	Surface fish (n=5) vs Pachón (n=4)	30-dpci	0.1	Post-hoc Welch’s test
**Fig. 6h**	BZ CM proliferation	Control (n=4) vs DEX (n=4)	14-dpci	0.0055	Two-sided Student's t-test
**Fig. 6i**	BZ CM proliferation	PBS (n=3) vs Clodronate (n=3)	14-dpci	0.6745	Two-sided Student's t-test
**Fig. 6j**	BZ CM proliferation	Control (n=3) vs ACA (n=4)	14-dpci	0.0506	Two-sided Student's t-test
**Fig. 7c**	IgM+ cells	Uninjured (n=6) vs 14-dpci (n=7)	14-dpci	0.0221	Two-sided Mann-Whitney U

**Fig. 7c**	IgM$^+$ cells	Uninjured (n=6) vs 21-dpci (n=8)	21-dpci	0.0168	Two-sided Mann-Whitney U
**Fig. 7f**	Wound size	wild-type (n=7) vs RAG (n=8)	21-dpci	0.0289	Two-sided Mann-Whitney U
**Fig. 7f**	Wound size	wild- type (n=8) vs RAG (n=8)	60-dpci	0.0006	Two-sided Mann-Whitney U
**Fig. 7g**	BZ CM proliferation	wild-type (n=3) vs RAG (n=3)	14-dpci	0.0261	Two-sided Student's t-test
**Sup. Fig. 3d**	*DiI uptake in macrophages*	*c1qa*$^+$ (n=3) vs *c1qa*$^+$DiI$^+$ (n=3)	14-dpci	0.700	Two-sided Mann-Whitney U
**Sup. Fig. 3h**	*cd37*$^+$ cells	PBS (n=3) vs Clodronate (n=3)	14-dpci	1	Two-sided Mann-Whitney U
**Sup. Fig. 3j**	*cd37*$^+$ cells	wild-type (n=4) vs Panther (n=4)	14-dpci	0.0686	Two-sided Mann-Whitney U
**Sup. Fig. 4b**	*cd37*$^+$ cells	Control (n=4) vs ACA (n=4)	30-dpci	0.342	Two-sided Mann-Whitney U
**Sup. Fig. 4e**	*cd37*$^+$ cells	Control (n=3) vs ACA (n=3)	30-dpci	0.498	Student's t-test
**Sup. Fig. 4g**	Wound size	Control (n=3) vs ACA (n=3)	30-dpci	0.069	Two-sided Student's t-test
**Sup. Fig. 4j**	*cd37*$^+$ cells	Control (n=4) vs ACA (n=4)	30-dpci	0.0618	Two-sided Welch's test
**Sup. Fig. 4l**	Wound size	Control (n=4) vs ACA (n=4)	30-dpci	0.1527	Two-sided Student's t-test
**Sup. Fig. 5d**	BZ CM proliferation	Control (n=4) vs ACA (n=5)	5-dpci	0.863	Two-sided Student's t-test
**Sup. Fig. 5e**	BZ CM proliferation	Control (n=3) vs ACA (n=3)	30-dpci	0.5613	Two-sided Student's t-test

Supplementary table 3| Key sources.

Reagent/ Antibody/ Kit	Source	Cat/ SKU/Identifier
	**Antibodies**	
Myosin Heavy Chain (MF20) Monoclonal Mouse	DSHB	AB_2147781
Proliferating Cell Nuclear Antigen (Pcna) Monoclonal Mouse	Dako	M0879
Rabbit polyclonal against Myocyte Enhancer Factor 2	Santa Cruz Biotechnology	SC-313
Alexa Fluor® 488	Invitrogen	A11001
Alexa Fluor® 555	Invitrogen	A31570
TSA plus Cy3	Perkin Elmer	NEL744001KT
TSA plus Cy5	Perkin Elmer	NEL745001KT
TSA plus FITC	Perkin Elmer	NEL756001KT
Alexa 488 Goat anti-mouse	Invitrogen	A55058
Alexa 546 Donkey anti-rabbit	Invitrogen	A10040
Chicken anti-GFP	Abcam	Ab13970
Goat anti-chicken 488	Thermo	A11039
	**Inhibitors**	
Dexamethasone	Sigma	D2915-100MG
DHMEQ	Med-Chem Express	HY-14645-5mg
Clodronate liposomes	Liposoma	CI-005-005
PBS liposomes	Liposoma	CI-005-005
PBS-DiL liposomes	Liposoma	CI-005-005
Acalabrutinib	Med-Chem Express	HY-17600
	**Chemicals**	

PEG300	Med-Chem Express	HY-Y0873-100mL
Corn oil	Med-Chem Express	8001-30-07
DMSO	ThermoFisher	022914
Saline	CD Medical	FS533
MS-222	Sigma	A5040
Phosphate Buffered Saline	Sigma	P4417
Xylene	Sigma	214736
4% paraformaldehyde in PBS	Santa Cruz	sc-281692
TNB block	Perkin Elmer	NEL702001KT
Butanol	Sigma	281549-1L
Ethanol	Sigma	51976
Paraffin wax	Sigma	327212
Histoclear	National diagnostics	AGR1353
Bouin’s solution	Sigma	HT10132
Methyl blue	Sigma	95290
Orange-G	Sigma	O7252
Acid fuchsin	Sigma	F8129
Mowiol 4-88	Sigma	81381
Dpx mountant	Sigma	06522-500ML
Phosphomolybdic acid	ThermoFisher	10590542
Prolong anti-gold antifade mountant	ThermoFisher	P36930
HCl	Sigma	SKU:320331-500ML
Sodium bicarbonate	Sigma	S5761

Heart digestion and sequencing

pluriStrainer® 100-µm	Pluriselect	43-50100-51
2,3-butanedione monoxime	Sigma	B0753
HEPES	Sigma	H3393
CaCl2	Sigma	C4901
Sheep serum	Sigma	S3772
Glucose	Sigma	G7528
Taurine	Sigma	T8691
Collagenase II	Gibco	17101-015
Collagenase IV	Gibco	17104-019
DNAse I	Sigma	10104159001
DMEM	Life Technologies	22320-022
Fetal Bovine Serum	ThermoFisher	A3840001
Liberase	Sigma	5401119001
HBSS	ThermoFisher	14175095
10x Chromium Chip B	10x Genomics	1000073
10x Chromium Next GEM Single Cell 3’ v3.1	10x Genomics	1000092
Illumina Nextseq® 500/550 High Output Kit	Illumina	FC-404-2005
Qubit RNA High Sensitivity Assay Kit V2	Invitrogen	Q32852
Nuclease-free water	ThermoFisher	AM9935
Quick-RNA MicroPrep kit	Zymo Research	R1051
QuantSeq 3' mRNA-Seq Library Prep Kit (FWD)	Lexogen	

	**RNAscope *in situ* hybridisation probes and kits**	
Ame-tnfa	ACD Biosciences, Biotechne	535021
Ame-nfkb2	ACD Biosciences, Biotechne	1052861-C3
Ame-c1qa	ACD Biosciences, Biotechne	814911-C2
Ame-ptprc	ACD Biosciences, Biotechne	874211-C2
Ame-cd37	ACD Biosciences, Biotechne	1052831-C1
Ame-mmp9	ACD Biosciences, Biotechne	532021- C1
Dr-cd37	ACD Biosciences, Biotechne	1295511-C2
RNAscope probe diluent	ACD Biosciences, Biotechne	300041
RNAscope Multiplex Fluorescent V2 Assay	ACD Biosciences, Biotechne	323100
	**Deposited data**	
bulk RNAseq data files of adult wild type zebrafish strains	Lekkos et al., 2025	GSE234990 (GSE234934)
3' UTR mRNAseq data files of adult *Astyanax mexicanus*	Lekkos et al., 2025	GSE234990 (GSE234989)
Single-cell RNA sequencing data files	This manuscript	GSE291684
	**Experimental models**	
Surface-dwelling *Astyanax mexicanus*	University of Oxford	GSE291684
Cave-dwelling *Astyanax mexicanus*	University of Oxford	GSE291684
EKW/TL	University of Bristol	ZDB-GENO-990520-2, ZDB-GENO-990623-2
Tg(*Cau.ighm:EGFP*)	University of Bristol	ZDB-TGCONSTRCT-131024-2
rag ^{hu1999}	University of Bristol	ZDB-ALT-090610-6
	**Software and algorithms**	
Astyanax mexicanus genome assembly	https://www.ensembl.org/Astyanax_mexicanus/	Mexican tetra (*Astyanax mexicanus*-2.0)
Astyanax mexicanus gene annotations	Ensembl v.103 release with extended 3'UTR[1]	GSE234989

Danio rerio genome assembly	ftp.ensembl.org:21/pub/release-104/fasta/danio_rerio/dna/Danio_rerio.GRCz11.dna_sm.primary_assembly.fa.gz	GRCz11/danRer11
Danio rerio gene annotation	ftp.ensembl.org/pub/release-104/gtf/danio_rerio/Danio_rerio.GRCz11.104.gtf.gz	Ensembl v.104
ImageJ	NIH	https://imagej.net/Fiji
GraphPad Prism v9.10	GraphPad Software Inc	https://www.graphpad.com/scientificsoftware/prism/
R (v.4.0.1)	R Core Team (2021)	https://www.R-project.org/.
DESeq2 1.44.0	[2]	
STAR (v.2.7.6a)	[3]	
Pheatmap (R package v1.0.8)	[4]	https://CRAN.R-project.org/package=pheatmap
gplots	[5]	https://CRAN.R-project.org/package=gplots
Seurat v3.2.2	[6,7]	https://github.com/satijalab/seurat
BisqueRNA 1.0.5	[7]	https://www.rdocumentation.org/packages/BisqueRNA/versions/1.0.5
Harmony 1.2.3	[8]	https://www.rdocumentation.org/packages/harmony/versions/2.0.5
Fgsea 1.30.0	[9]	https://bioconductor.org/packages/release/bioc/html/fgsea.html
Ggpubr 0.6.0	[10]	https://CRAN.R-project.org/package=ggpubr
Msigdbr 7.5.1	[11]	https://github.com/igordot/msigdbr/
Biobase 2.64.0	[12]	https://www.bioconductor.org/packages/release/bioc/html/Biobase.html
scProportionTest	[13]	https://github.com/rpolicastro/scProportionTest

Equipment

Confocal microscope	Zeiss	LSM770
Microscope	Nikon Instruments Inc	ECLIPSE Ci

Stereoscope	Olympus	SZ61
Microtome	Leica	Leica EM UC7
QubitTM 3 Fluorometer	Invitrogen	Q33216
Nanodrop 2000	ThermoFisher	Microm HM325
RNase-free tubes	Ambion	AM12450
pestles	VWR	431-0094
Pellet Mixer	VWR	431-0100
SuperFrost glass slides	VWR	631-0108
96-well plate	ThermoFisher	AB-0800
Sealer	Thermo Scientific	AB-0558
