# Supplementary material for: Absence of a prolonged macrophage and B cell response inhibits heart regeneration in the Mexican cavefish
Source: Nat Commun. 2026 Sep 17;17:10922. doi: 10.1038/s41467-026-77725-4 (PMC13680611; doi:10.1038/s41467-026-77725-4)
Supplement: Supplementary file 2 [file 41467_2026_77725_MOESM2_ESM.pdf]

nature portfolio

Peer Review File

Absence of a prolonged macrophage and B cell response inhibits heart regeneration in the Mexican cavefish

Corresponding Author: Dr Mathilda Mommersteeg

This file contains all reviewer reports in order by version, followed by all author rebuttals in order by version.

Version 0:

Reviewer comments:

Reviewer #1

(Remarks to the Author)
Summary:
By comparing the immune responses of two Mexican cavefish morphs after heart injury, the authors of this manuscript demonstrate that prolonged infiltration of leukocytes, especially the macrophage and B cell activity, is crucial for heart regeneration. Surface-dwelling fish, which are capable of heart regeneration, maintain a relatively high number of phagocytic macrophages during the late stages of regeneration. By depleting these macrophages between 7 and 14 days post-cryo-injury (dpci), the authors show that macrophages help to reduce scarring by inhibiting fibroblast proliferation, despite single-cell data suggesting these macrophages express genes promoting fibroblast growth. Interestingly, the authors also found that B cells are initially scarce in both morphs but expand significantly in surface fish after 7 dpci, peaking at 30 dpci. Gene enrichment analysis links increased B cell numbers to proliferation events. Depleting B cells with Dexamethasone (DEX) and Acalabrutinib (ACA) from 7 to 14 dpci reduces cardiomyocyte proliferation, whereas macrophage depletion and early B cell depletion do not. Finally, in zebrafish, B cells show a conserved role in regeneration, as rag1 mutants display larger injuries and reduced cardiomyocyte proliferation at late stages. However, the prior reporting of B cells' role in heart regeneration limits the novelty of this manuscript.

This manuscript provides new insight into the late-stage roles of macrophages in suppressing fibroblast proliferation. They also extend previous findings by demonstrating that B cells primarily function at late stages of heart injury to promote cardiomyocyte proliferation. However, the manuscript lacks sufficient data to support its conclusions and does not explore its new insights in depth. Below are my major comments and minor comments for this manuscript.

Major comments:
1. Depletion of macrophages using clodronate liposomes (CL) significantly impairs regeneration, which the authors suggest may be partly due to the macrophages' ability to inhibit fibroblast proliferation. They also propose that late-stage macrophages are phagocytic and help reduce scarring through phagocytosis. The relationship between these two mechanisms should be further investigated. To address this, two suggestions are proposed. Firstly, after depleting macrophages with clodronate liposomes, the authors should verify whether they specifically depleted the phagocytic macrophage population or whether the inhibitory effect on fibroblast proliferation might originate from non-phagocytic macrophages. They could quantify the number of remaining macrophages after CL treatment and compare it with results from DiI liposome injection experiment or co-inject DiI liposomes with CL and assess whether the remaining macrophages retain phagocytic activity by monitoring DiI uptake. Second, they could use pharmacological inhibitors to block macrophage phagocytosis and examine whether this leads to impaired regeneration and increased fibroblast proliferation. The presence of phagocytic activity in late-stage macrophages does not necessarily imply that these macrophages regulate the regeneration process through phagocytosis. Additionally, the authors should present quantitative data alongside image-based results to more robustly support their conclusions.
2. The GO analysis results show that late-stage macrophages are enriched for genes related to 'positive regulation of fibroblast proliferation,' which appears contradictory to the findings from their depletion experiments. The authors should address and discuss this inconsistency.
3. Zebrafish studies suggest that cardiomyocyte proliferation begins at an early developmental stage but significantly decreases by later stages, around 10 days post-injury (10dpi). It would be beneficial for this study to illustrate the dynamic changes in cardiomyocyte proliferation throughout the process in their models, emphasizing the continued importance of cardiomyocyte proliferation at later stages.

4. The number of B cells increases after injury, beginning at 7 dpci and peaks at 30 dpci. In this manuscript, B cells are depleted from 7 to 11/14 dpci, and effects are evaluated at 14 and 30 dpci. Two aspects require further investigation. First, given the much higher B cell count at 30 dpci, it is necessary to determine whether depletion during 7–11 dpci also reduces B cell numbers at that time point, which would help clarify the timing of B cell involvement in heart regeneration. Second, depleting B cells at later stages (24- 30dpi) would help establish whether late-stage B cells contribute to regeneration and regulate cardiomyocyte proliferation.
5. When treating fish with Acalabrutinib at 2, 3, and 4 dpci, the authors should also examine whether early B cell depletion affects cardiomyocyte proliferation at later stages and impacts the overall regeneration outcome. It helps to explore whether the B cells present at later stages are recruited from an early population or are newly generated during the late regeneration process.

Minor comments:
1. In the sentence from lines 78 to 81 ('The cavefish have evolved... exposure'), using 'the cavefish' as the subject is confusing, as it could also refer to the Mexican cavefish. It would be clearer to use 'the cave-dwelling fish' instead.
2. In line 164, the manuscript claims that neutrophils peak at 3 dpi. However, both the scRNA-seq data and RNAscope staining targeting mmp9 suggest that neutrophils actually peak at 1 dpi.
3. In Figure 5i, the title 'Enrichment of B cell scores in bulk RNA-seq' is misleading. According to lines 361–363, the enrichment analysis was performed using genes correlated with B cell scores, not the scores themselves.
4. In Figure 5f, the axis differs from the other figures. For consistency, it would be better to revise it to match the formatting used throughout the manuscript.
5. The sample size in several experiments throughout the manuscript is too small to draw strong conclusions. This is particularly evident in Supplementary Figure 3, where Figure 3c shows a decreasing trend that does not reach statistical significance, likely due to limited sample size. The authors should increase sample sizes, especially for key comparisons, and clearly label p-values in their quantitative data to improve clarity and rigor.

Reviewer #2

(Remarks to the Author)
The study by Sengul et al. investigates the contrasting heart regeneration abilities of two ecotypes of the Mexican cavefish, Astyanax mexicanus: the surface-dwelling fish, which can regenerate heart tissue, and the cave-dwelling Pachón cavefish, which forms scar tissue instead. The research utilizes single-cell transcriptomic data collected at various time points following cryoinjury to the fish heart in both ecotypes.

The authors identify differences in the activity of both innate and adaptive immune cells between the injured cavefish and surface fish, highlighting their potential role in modulating regeneration. Notably, in regenerative surface fish, heart injury triggers a prolonged, balanced immune response involving both myeloid (e.g., macrophages) and lymphoid (e.g., B cells) populations. In contrast, Pachón cavefish exhibit a rapid but short-lived immune response, leading to scar formation rather than regeneration. A sustained immune presence in surface fish was confirmed via in situ hybridization for leukocyte markers, and by experimental approaches like suppression of inflammation with Dexamethasone from 7 to 14 days post injury (peak time points of elevated immune cell activity), led to larger scars and poorer regeneration, underlining the necessity of prolonged immune activity.

A major concern with this paper is the lack of biological replicates, using a single fish per time point, raises concerns about individual variation vs real biological differences, in other words, how complete is the data to reach the conclusions presented in this study. The authors should support the quality of each dataset per individual fish to ensure that, despite the lack of repeatability, the data possesses the necessary quality. For instance, if the contradiction between scRNA and RNA scope for the neutrophil profile is biological and not methodological. Besides, the quality of the scRNA analysis can be improved, as exemplified by the labeling of doublets and dying cells in different scRNA plots shown across the manuscript. Furthermore, while the focus is on immune cell populations, analyzing cell states in cardiomyocytes, essential for heart regeneration, is missing. The bioinformatic analysis should also be more detailed, and the features of the specimens used should be clearly described to allow reproducibility. This is particularly important because the evidence presented contrasts with patterns observed in the non-heart regenerating medaka fish and the heart-regenerating zebrafish.

If the authors can demonstrate that, even in the absence of biological replicates and likely individual variation, the data remains robust, this paper could significantly contribute to our understanding of the pathways for heart regeneration.

Finally, the study underscores how the fine-tuning of immune responses is related to various disease phenotypes, emphasizing the need for new models to study regeneration versus non-regeneration (scar formation). In particular, the cavefish, with its evolved metabolic and immune-enhanced capacities in the caves, serves as an impressive model for human-related diseases.

Detailed Comments:

• Line 32: Complete the sentence with "Pachón population." For readers who are not familiar with the cavefish system, merely mentioning "Pachón" is non-informative.

• Line 78: Be more precise by briefly mentioning 'at least one cavefish population,' given that only another two populations

have been tested for heart regeneration capacities (one cavefish and one hybrid population), out of the 35 cavefish populations known to date.

• Line 79–80: Consider adding the appropriate reference for this statement.

• Line 81: Instead of "reduced pathogen exposure," consider "changes in pathogen exposure"; some studies have shown still high prevalences of parasites in certain caves, including Pachón.

• Line 108: Specify whether the cell types were as expected according to zebrafish or reference a previous study. As stated, it sounds ambiguous.

• Figure 2a: In the subclustering for cell assignments, some cells appear labeled as doublets, which should have been removed during filtering steps. Many pipelines are available for this procedure, which should be applied for your dataset.

• Figure 3a-d: Highly uninformative, as genes are not presented. Consider moving these figures to the supplementary materials.

• Line 618: Provide more details about the specimens used. I would expect the use of age-matched fish for all these experiments, as well as considerations for sex and/or size. Immune system maturation (though still not tested yet in A. mexicanus) likely changes with age and differs between sexes, therefore, affecting regeneration/scar-formation capabilities.

• Line 655 “Single-cell RNA sequencing and analysis”:
1. Double-check the spelling and proper writing of this section; many errors are found.
2. For the cell suspension preparation, the authors used 100 µm filters. scRNA droplet-based methods like 10X have a size limitation of 30 µm. The authors ideally should have optimized the cell preparation for such sequencing goals; the use of such a cell suspension could have caused major issues in data quality. Therefore, the authors should mention THE QUALITY AND CELL VIABILITY THRESHOLD used before sequencing.
3. In the supplementary data, the number of cells and quality per sample should be stated to ensure the reader that quality was considered for each time point/sample-fish, and not just the final number of cells in the integrated dataset.
4. Considering, as mentioned at the beginning of this report, the lack of biological replicates. Also, in the supplementary materials, mention all the markers used for cell assignment, not just a few as in the plot Fig S1b, to ensure reproducibility.
5. As mentioned before, authors should implement doublet filtering, and even consider tools like CellBender to improve the quality of the data.

• Line 678: Missing mention of the number of individuals/replicates per treatment, for this and all the following experiments.

• Line 781: Were different genome versions used for scRNA-seq and bulk RNA-seq, versions two and three, respectively? If so, both analyses should implement the same reference genome.

Discussion:
The authors are missing key points or open questions and future directions; including those related to what the adaptive component of scar formation is in this particular cave and not in others of the few analyzed.

Line 1070: The presence of dead/dying cells as one of the clusters raises a huge concern, since you are still showing contamination in your data. In the methods, you stated removal of this ambient contamination, but it is still displayed on cell populations labeled

Version 1:

Reviewer comments:

Reviewer #1

(Remarks to the Author)
All my conerns have been addressed in the revised manuscript. I have no further question.

Open Access This Peer Review File is licensed under a Creative Commons Attribution 4.0 International License, which permits use, sharing, adaptation, distribution and reproduction in any medium or format, as long as you give appropriate credit to the original author(s) and the source, provide a link to the Creative Commons license, and indicate if changes were made.
In cases where reviewers are anonymous, credit should be given to 'Anonymous Referee' and the source.
The images or other third party material in this Peer Review File are included in the article's Creative Commons license, unless indicated otherwise in a credit line to the material. If material is not included in the article's Creative Commons license and your intended use is not permitted by statutory regulation or exceeds the permitted use, you will need to obtain permission directly from the copyright holder.
To view a copy of this license, visit https://creativecommons.org/licenses/by/4.0/

Response to reviewer comments

We thank both reviewers for their careful reading and for their constructive feedback. Their comments substantially strengthened the study. We provided a point-by-point response to each comment. All changes made to the manuscript are highlighted in yellow.

Reviewer #1 (Remarks to the Author):

Summary:
By comparing the immune responses of two Mexican cavefish morphs after heart injury, the authors of this manuscript demonstrate that prolonged infiltration of leukocytes, especially the macrophage and B cell activity, is crucial for heart regeneration. Surface-dwelling fish, which are capable of heart regeneration, maintain a relatively high number of phagocytic macrophages during the late stages of regeneration. By depleting these macrophages between 7 and 14 days post-cryo-injury (dpci), the authors show that macrophages help to reduce scarring by inhibiting fibroblast proliferation, despite single-cell data suggesting these macrophages express genes promoting fibroblast growth. Interestingly, the authors also found that B cells are initially scarce in both morphs but expand significantly in surface fish after 7 dpci, peaking at 30 dpci. Gene enrichment analysis links increased B cell numbers to proliferation events. Depleting B cells with Dexamethasone (DEX) and Acalabrutinib (ACA) from 7 to 14 dpci reduces cardiomyocyte proliferation, whereas macrophage depletion and early B cell depletion do not. Finally, in zebrafish, B cells show a conserved role in regeneration, as rag1 mutants display larger injuries and reduced cardiomyocyte proliferation at late stages. However, the prior reporting of B cells' role in heart regeneration limits the novelty of this manuscript.

This manuscript provides new insight into the late-stage roles of macrophages in suppressing fibroblast proliferation. They also extend previous findings by demonstrating that B cells primarily function at late stages of heart injury to promote cardiomyocyte proliferation. However, the manuscript lacks sufficient data to support its conclusions and does not explore its new insights in depth. Below are my major comments and minor comments for this manuscript.

We thank Reviewer #1 for the thorough and constructive summary of the manuscript and for identifying key areas that required experimental support. We address each major and minor comment in turn below. In brief, we have performed additional phagocytosis experiments including pharmacological inhibition with 2-acetamidophenol (AMP) and re-mapped our scRNA-seq data to the updated AstMex3 genome; generated a full cardiomyocyte proliferation timeline after cryoinjury; carried out the requested late-stage Acalabrutinib experiment (26–30 dpci) and the early-treatment proliferation analysis; and increased sample sizes for several key comparisons. We hope these extensive additions address the reviewer's concerns.

Major comment #1:

Depletion of macrophages using clodronate liposomes (CL) significantly impairs regeneration, which the authors suggest may be partly due to the macrophages' ability to inhibit fibroblast proliferation. They also propose that late-stage macrophages are phagocytic and help reduce scarring through phagocytosis. The relationship between these two mechanisms should be further investigated. To address this, two suggestions are proposed. Firstly, after depleting macrophages with clodronate liposomes, the authors should verify whether they specifically depleted the phagocytic macrophage population or whether the inhibitory effect on fibroblast proliferation might originate from non-phagocytic macrophages. They could quantify the number of

remaining macrophages after CL treatment and compare it with results from DiI liposome injection experiment or co-inject DiI liposomes with CL and assess whether the remaining macrophages retain phagocytic activity by monitoring DiI uptake. Second, they could use pharmacological inhibitors to block macrophage phagocytosis and examine whether this leads to impaired regeneration and increased fibroblast proliferation. The presence of phagocytic activity in late-stage macrophages does not necessarily imply that these macrophages regulate the regeneration process through phagocytosis. Additionally, the authors should present quantitative data alongside image-based results to more robustly support their conclusions.

We thank the reviewer for these excellent and well-targeted suggestions. We have now addressed both points with new experiments. After using Clodronate liposomes, the number of macrophages were very low (Fig.4d), confirming our findings that the majority of macrophages at these late stages are phagocytotic. We co-injected Clodronate with DiI-PBS liposomes and found that DiI uptake was significantly reduced in co-injected fish compared to PBS controls (Fig. 4e–f, Supplementary Fig. 3b), confirming that the surviving macrophages have lost or severely compromised phagocytic function.

Regarding the second suggestion, we followed reviewer's recommendation and used a pharmacological inhibitor of phagocytosis, 2-Acetamidophenol, and co-injected with DiI liposomes from 7- to 14-dpci (Fig. 4k). AMP treatment did not affect macrophage numbers in the wound (Fig. 4l, Supplementary Fig. 3g) but did affect their ability to take up DiI-liposomes. (AMP-treated fish showed significantly reduced DiI liposome uptake compared to controls (Fig. 4m–n), confirming effective *in vivo* phagocytosis inhibition. Critically, AMP-treated hearts exhibited significantly elevated epicardial transgelin (TAGLN, which is a marker for (myo)fibroblasts) levels at 14 dpci (Fig. 4o–p) and significantly larger wounds at 30 dpci (Fig. 4r–s). Together, the Clodronate and AMP data robustly demonstrate that it is specifically the phagocytic function of late-stage macrophages. Quantitative data accompany all image-based results throughout the revised manuscript.

Specifically:
Lines 291–312: Clodronate liposome experiments
Lines 293–301: Co-injection of Clodronate + DiI-PBS and uptake quantification (Fig. 4e–f)
Lines 313–323: AMP pharmacological inhibition experiment (Fig. 4k–s)
Lines 308–312: Macrophage/B cell independence and Panther zebrafish mutant

Major comment #2:

The GO analysis results show that late-stage macrophages are enriched for genes related to 'positive regulation of fibroblast proliferation,' which appears contradictory to the findings from their depletion experiments. The authors should address and discuss this inconsistency.

We thank the reviewer for highlighting this. We re-mapped all our scRNAseq reads to the latest updated A. mexicanus genome assembly (AstMex3) with improved gene annotations. This substantially improved data quality (Supplementary Fig. 6) and importantly, affected the gene-level pathway enrichment results. In the re-analysis, 'positive regulation of fibroblast proliferation' is no longer among the top enriched pathways in late-stage surface fish macrophages. The top enriched pathway is now lysososome, which is fully consistent with an active phagocytic state. GO term enrichment now highlights lysosome, cell migration, endocytosis demonstrating that late-stage macrophages function primarily through phagocytosis. The previous inconsistency arose from mapping artifacts in the older genome

version. And revised analysis resolved this contradiction entirely. Therefore, we decided not to explore a role in regulating proliferation anymore but focused on the phagocytosis differences. As already highlighted after major comment 1, quantification of scarring in the hearts after inhibition of phagocytosis highlighted the role of the late macrophages during regeneration. We have updated the relevant text, figures, and the methods accordingly.

Lines 283–285: GO term analysis now shows lysosome as top pathway (Fig. 4b)

Major comment #3:

Zebrafish studies suggest that cardiomyocyte proliferation begins at an early developmental stage but significantly decreases by later stages, around 10 days post-injury (10dpi). It would be beneficial for this study to illustrate the dynamic changes in cardiomyocyte proliferation throughout the process in their models, emphasizing the continued importance of cardiomyocyte proliferation at later stages.

The reviewer mentions cardiomyocyte proliferation from an early developmental stage until 10dpi. Based on the rest of the comment, we have interpretted 'early developmental stage' as 'early stage after injury' in this context. We have now generated an extensive timeline of border zone cardiomyocyte proliferation after cryoinjury in both surface fish and Pachón, including uninjured, 3-, 7-, 14-, and 30-dpci (Fig. 6e-f). We had previously characterised a proliferation timeline after resection injury, where proliferation peaks at 7-dpci without significant differences between morphs; however, this was not performed after cryoinjury.

Our new cryoinjury data confirms the slightly higher levels of proliferation at 7-dpci in surface, which however remain insignificant from cavefish levels. What is different from the resection data is that proliferation remains high at 14-dpci and even 30-dpci. This data fits very nicely with our finding of a role for B cells at these late timepoints as well as with the prolonged regeneration period seen after cryo-injury compared to resection injury.

Although PCNA protein levels are similar between morphs at most timepoints consistent to prior resection injury data, single-cell mRNA-level cell cycle analysis reveals that Pachón cardiomyocytes become arrested in S-phase without progressing to M-phase, retaining PCNA protein expression but failing to complete mitosis (Fig. 6c–d). This temporal profile aligns precisely with the window of B cell recruitment (from 7 dpci), directly supporting our conclusion that B cells promote cell cycle progression into mitosis at late stages, and this extended timeline data is now presented in Fig. 6f.

Lines 443–477: Full cardiomyocyte sub-clustering and cell cycle analysis
Lines 458–462: S-phase and M-phase dynamics across timepoints (Fig. 6d)
Lines 465–477: PCNA/Mef2 immunostaining timeline (Fig. 6e–f)

Major comment #4:

The number of B cells increases after injury, beginning at 7 dpci and peaks at 30 dpci. In this manuscript, B cells are depleted from 7 to 11/14 dpci, and effects are evaluated at 14 and 30 dpci. Two aspects require further investigation. First, given the much higher B cell count at 30 dpci, it is necessary to determine whether depletion during 7–11 dpci also reduces B cell numbers at that time point, which would help clarify the timing of B cell involvement in heart regeneration. Second, depleting B cells at later stages (24- 30dpi) would help establish whether late-stage B cells contribute to regeneration and regulate cardiomyocyte proliferation.

We thank the reviewer for these important points, both of which we have now addressed experimentally.

Regarding the first point, we now included quantification of *cd37*⁺ B cell numbers in the wound at 30-dpci in fish treated with Acalabrutinib within the main treatment window (Supplementary Fig. 4a–b). B cell numbers at 30-dpci were not significantly different from controls in this cohort (Supplementary Fig. 4b, p = 0.342), indicating that the early inhibition window does not cause long-term depletion of B cells at 30-dpci. This clarifies that the effect on wound size at 30-dpci (Fig. 5f) is a consequence of impaired B cell function/signalling during the 7–14-dpci window specifically, rather than sustained B cell depletion. This is an important mechanistic distinction as it supports the idea that B cells exert a critical time-limited paracrine effect on cardiomyocyte proliferation rather than a chronic structural role.

Regarding the second point, we have now performed the late-stage inhibition experiment, treating fish with Acalabrutinib from 26- to 30-dpci (Supplementary Fig. 4h–l). This treatment showed a non-significant trend towards reduced B cell numbers (p = 0.062) and a non-significant trend towards larger wounds (p = 0.153) at 30-dpci. We discuss this in the revised manuscript: the absence of statistical significance is most likely attributable to the very short treatment window (only 4 days from onset of inhibition to heart collection), which may be insufficient to deplete B cells already present and disrupt ongoing B cell–cardiomyocyte interactions. A longer treatment window would be expected to produce a stronger effect. These data are presented in Supplementary Fig. 4h–l and discussed in the main text.

Lines 373–385: Acalabrutinib treatment windows and results
Lines 380–385: Late-stage (26–30 dpci) treatment discussion (Supplementary Fig. 4h–l)

Major comment #5:

When treating fish with Acalabrutinib at 2, 3, and 4 dpci, the authors should also examine whether early B cell depletion affects cardiomyocyte proliferation at later stages and impacts the overall regeneration outcome. It helps to explore whether the B cells present at later stages are recruited from an early population or are newly generated during the late regeneration process.

We thank the reviewer for this important suggestion. We have now included a full analysis of this early treatment cohort (Supplementary Fig. 4c–g and Supplementary Fig. 5c–e). Fish treated with Acalabrutinib from 2 to 5 dpci (prior to meaningful B cell recruitment) showed no significant difference in *cd37*⁺ B cell numbers at 30-dpci (Supplementary Fig. 4e, p = 0.510), no significant difference in wound size at 30-dpci (Supplementary Fig. 4g, p = 0.069), and no significant difference in border zone cardiomyocyte proliferation at either 5-dpci (Supplementary Fig. 5d, p = 0.863) or 30-dpci (Supplementary Fig. 5e).

These data make two important points: first, they confirm that Acalabrutinib does not have direct off-target effects on cardiomyocyte proliferation, strengthening our conclusion that the effect of 7–11 dpci treatment is specifically mediated through B cell inhibition. Second, and more biologically significant, the absence of an effect on B cell numbers at 30-dpci strongly suggests that the late B cell population is not derived from an early (pre-7 dpci) population that is susceptible to early Acalabrutinib treatment. Rather, the late B cells appear to be freshly recruited after 7-dpci, consistent with our scRNA-seq data showing minimal B cell numbers in both morphs at early timepoints (Fig. 2e). This is now discussed in the revised main text.

Lines 486–492: Early Acalabrutinib (2–5 dpci) confirms no off-target effects (Supplementary Figs. 4c–g, 5c–e)

Minor comments:

1. In the sentence from lines 78 to 81 ('The cavefish have evolved... exposure'), using 'the cavefish' as the subject is confusing, as it could also refer to the Mexican cavefish. It would be clearer to use 'the cave-dwelling fish' instead.

Lines 76–80: Changed "the cavefish" to "the cave-dwelling fish"

2. In line 164, the manuscript claims that neutrophils peak at 3 dpi. However, both the scRNA-seq data and RNAscope staining targeting mmp9 suggest that neutrophils actually peak at 1 dpi.

Lines 176–179: Neutrophil peak corrected to 1-dpci

3. In Figure 5i, the title 'Enrichment of B cell scores in bulk RNA-seq' is misleading. According to lines 361–363, the enrichment analysis was performed using genes correlated with B cell scores, not the scores themselves.

The figure title has been updated to 'Pathways Enriched in Genes Correlated with B cell Scores', which accurately reflects that the WikiPathways enrichment was performed on the ranked gene list derived from Spearman correlation with B cell deconvolution scores, not on the scores themselves. Lines 432–433

4. In Figure 5f, the axis differs from the other figures. For consistency, it would be better to revise it to match the formatting used throughout the manuscript.

The y-axis of Figure 5f has been updated to display wound size as '%scar/ventricle size', consistent with the axis format used in equivalent panels throughout the manuscript. Fig. 5f y-axis, Line 426–427

5. The sample size in several experiments throughout the manuscript is too small to draw strong conclusions. This is particularly evident in Supplementary Figure 3, where Figure 3c shows a decreasing trend that does not reach statistical significance, likely due to limited sample size. The authors should increase sample sizes, especially for key comparisons, and clearly label p-values in their quantitative data to improve clarity and rigor.

We agree with the reviewer. We have increased n-numbers for several key experiments in the revised manuscript; specific instances where sample sizes have been augmented are highlighted in the revised text and figure legends. Regarding Supplementary Figure 3 (DHMEQ experiment's B cell and macrophage quantification after NF-κB inhibition): as we discuss in the manuscript, NF-κB inhibition does not significantly affect regeneration, and this figure was therefore not central to our main conclusions. To avoid unnecessary use of animals in experiments that do not substantively advance the key findings, we have removed this supplementary figure from the revised manuscript. All p-values are now clearly labelled on quantitative panels throughout, including for non-significant comparisons where relevant.

Lines 157–163: n numbers updated in figure legends throughout; DHMEQ supplementary figure removed.

Reviewer #2 (Remarks to the Author):

The study by Sengul et al. investigates the contrasting heart regeneration abilities of two ecotypes of the Mexican cavefish, Astyanax mexicanus: the surface-dwelling fish, which can regenerate heart tissue, and the cave-dwelling Pachón cavefish, which forms

scar tissue instead. The research utilizes single-cell transcriptomic data collected at various time points following cryoinjury to the fish heart in both ecotypes.

The authors identify differences in the activity of both innate and adaptive immune cells between the injured cavefish and surface fish, highlighting their potential role in modulating regeneration. Notably, in regenerative surface fish, heart injury triggers a prolonged, balanced immune response involving both myeloid (e.g., macrophages) and lymphoid (e.g., B cells) populations. In contrast, Pachón cavefish exhibit a rapid but short-lived immune response, leading to scar formation rather than regeneration. A sustained immune presence in surface fish was confirmed via in situ hybridization for leukocyte markers, and by experimental approaches like suppression of inflammation with Dexamethasone from 7 to 14 days post injury (peak time points of elevated immune cell activity), led to larger scars and poorer regeneration, underlining the necessity of prolonged immune activity.

A major concern with this paper is the lack of biological replicates, using a single fish per time point, raises concerns about individual variation vs real biological differences, in other words, how complete is the data to reach the conclusions presented in this study. The authors should support the quality of each dataset per individual fish to ensure that, despite the lack of repeatability, the data possesses the necessary quality. For instance, if the contradiction between scRNA and RNA scope for the neutrophil profile is biological and not methodological. Besides, the quality of the scRNA analysis can be improved, as exemplified by the labeling of doublets and dying cells in different scRNA plots shown across the manuscript. Furthermore, while the focus is on immune cell populations, analyzing cell states in cardiomyocytes, essential for heart regeneration, is missing. The bioinformatic analysis should also be more detailed, and the features of the specimens used should be clearly described to allow reproducibility. This is particularly important because the evidence presented contrasts with patterns observed in the non-heart regenerating medaka fish and the heart-regenerating zebrafish.

We thank Reviewer #2 for the detailed and constructive critique. The reviewer raised important concerns regarding single-fish scRNA-seq replication, scRNA-seq data quality (doublets, dying cells), missing biological details in the methods, and the bioinformatic analysis. We have addressed these comprehensively, as detailed below.

All scRNA-seq reads have been re-mapped to the updated AstMex3 genome with improved gene annotations and mitogenome appendance (see Supplementary Fig. 7 and updated Methods). This re-mapping substantially improved per-cell detection rates and removed spurious clusters. Strict doublet removal was performed using scDblFinder prior to downstream analysis, and ambient RNA contamination was removed using decontX. Updated Figure 1b shows that this has led to a strongly reduced number of clusters, with especially removal of the double-identify clusters that were likely doublets and dying-cell clusters that were present in the previous analysis, as shown in the updated Fig. 1b and Supplementary Fig. 7c. Critically, per-sample QC metrics (nCount_RNA, nFeature_RNA, mitochondrial content) before and after filtering are now presented in Supplementary Fig. 7a–b, and per-sample cell counts are shown in Supplementary Fig. 7d–e, enable assessing data quality at each individual time point and morph. All bioinformatic parameters used for QC are detailed in the revised Methods section, Methods Lines 754–755.

Lines 109–115: Updated cell count from 85,516 to 60,598 (Fig. 1b legend, Lines 136–140)
Lines 760–773: Updated bioinformatics methods (genome re-mapping, doublet removal, decontX)

We have now included a detailed re-clustering of cardiomyocytes (Fig. 6a–d and Supplementary Fig. 6a–b), identifying ten sub-populations including four wound border zone clusters, one of which (BZ4) is characterised by M-phase activity. Cell cycle analysis of these sub-populations reveals a progressive increase in cycling cardiomyocytes in surface fish from 3 dpci onwards that is absent in Pachón, directly linking immune cell dynamics to cardiomyocyte proliferation and providing the cardiomyocyte-level analysis the reviewer requested.

The difference in significance of neutrophil numbers between the scRNAseq and RNAscope data still remains and we acknowledge this in the revised Discussion. scRNA-seq was performed on whole ventricles, whereas RNAscope quantification targeted cells specifically within the wound region. Neutrophils that infiltrate but rapidly disperse throughout the ventricle, or that are present in the remote tissue would be captured by scRNA-seq but not by wound normalised RNAscope counts. The overall trend is consistent between methods, and this discrepancy reflects a known technical difference in spatial context rather than a true biological inconsistency.

If the authors can demonstrate that, even in the absence of biological replicates and likely individual variation, the data remains robust, this paper could significantly contribute to our understanding of the pathways for heart regeneration. Finally, the study underscores how the fine-tuning of immune responses is related to various disease phenotypes, emphasizing the need for new models to study regeneration versus non-regeneration (scar formation). In particular, the cavefish, with its evolved metabolic and immune-enhanced capacities in the caves, serves as an impressive model for human-related diseases.

We thank Reviewer #2 for appreciating the value and impact of the work in this manuscript.

Detailed Comments:

• Line 32: Complete the sentence with "Pachón population." For readers who are not familiar with the cavefish system, merely mentioning "Pachón" is non-informative.

This has been changed as suggested by the reviewer. Line 32.

• Line 78: Be more precise by briefly mentioning 'at least one cavefish population,' given that only another two populations have been tested for heart regeneration capacities (one cavefish and one hybrid population), out of the 35 cavefish populations known to date.

This has also been changed as suggested. Line 77: "at least one cavefish population" added.

• Line 79–80: Consider adding the appropriate reference for this statement.

The appropriate references have now been added to support this statement. Lines 78–79.

• Line 81: Instead of "reduced pathogen exposure," consider "changes in pathogen exposure"; some studies have shown still high prevalences of parasites in certain caves, including Pachón.

This has been changed as suggested. Line 80.

• Line 108: Specify whether the cell types were as expected according to zebrafish or reference a previous study. As stated, it sounds ambiguous.

The sentence has been clarified with a specific reference to canonical marker genes established in zebrafish and validated in previous A. mexicanus studies. An appropriate reference has been added.

Lines 110–111: Reference to canonical zebrafish markers added

• Figure 2a: In the subclustering for cell assignments, some cells appear labeled as doublets, which should have been removed during filtering steps. Many pipelines are available for this procedure, which should be applied for your dataset.

We thank the reviewer for flagging this. As noted in our general response, we have now re-mapped all reads to AstMex3 and applied strict doublet removal using scDblFinder prior to any downstream analysis. In some of our studies on zebrafish heart regeneration, we found that several cell-types obtain a dual cell-type identity during regeneration, for example, endocardial cells obtain expression profiles of myofibroblasts and hematopoietic cells, while still retaining their endocardial identity. This has also been reported in other cell-types in other papers. This was the reason we used less stringent cut-offs during our first analysis of the scRNAseq data used in the previous version of our manuscript, as we wanted to avoid removing possible double-identity populations. However, we realise that these are not important for the current study and we have now remapped the reads to the new genome and applied strict doublet removal. This has removed the ‘dual-identity’ populations as well as the dying cell population. We have updated Fig. 1b and Supplementary Fig. 7c–d reflect these improvements. The full bioinformatic pipeline including doublet removal parameters is described in the revised Methods section.

Lines 761–768: Full doublet removal pipeline described; Fig. 1b updated.

• Figure 3a-d: Highly uninformative, as genes are not presented. Consider moving these figures to the supplementary materials.

We have now added gene annotation to these heatmaps. The aim for showing these heatmaps is to highlight the overall different patterns of the most highly expressed genes over time. To show the differences between surface and cavefish, but also especially to highlight that the cells express other top markers at different timepoints in surface fish, whereas cavefish express similar genes at all stages.

Lines 228–234: Gene annotations added to heatmaps

• Line 618: Provide more details about the specimens used. I would expect the use of age-matched fish for all these experiments, as well as considerations for sex and/or size. Immune system maturation (though still not tested yet in A. mexicanus) likely changes with age and differs between sexes, therefore, affecting regeneration/scar-formation capabilities.

We thank the reviewer for raising this important point. The Animal Husbandry section of the Methods has been expanded. Adults aged 1–2 years were used consistently across all experiments, and age-matched wild-type fish served as controls in all zebrafish experiments. Mixed-sex cohorts were used to minimise potential sex-related bias. Lines 711–729.

• Line 655 “Single-cell RNA sequencing and analysis”:

1. Double-check the spelling and proper writing of this section; many errors are found.

The single-cell RNA-seq methods section has been thoroughly revised and corrected.

Lines 747–773: Methods section rewritten.

2. For the cell suspension preparation, the authors used 100 µm filters. scRNA droplet-based methods like 10X have a size limitation of 30 µm. The authors ideally should have optimized the cell preparation for such sequencing goals; the use of such a cell suspension could have caused major issues in data quality. Therefore, the authors should mention THE QUALITY AND CELL VIABILITY THRESHOLD used before sequencing.

We had selected the 100um filter after careful consideration. In a scRNAseq experiment we had done just previously, we had lost almost all cardiomyocytes.. Adult cardiomyocytes can reach ~125 µm in length (Kannan et al., 2019, https://doi.org/10.1101/654954), At the time of our experiments, no published studies had successfully captured intact cardiomyocytes using droplet-based scRNA-seq as well-recognised in the field (Palmer et al., 2024, https://doi.org/10.1161/CIRCRESAHA.124.323672 (2024)). Testing of different filter sizes showed that using the 100um filter was the only option to obtain reasonable cardiomyocyte numbers in the single cell suspension. As we realised that using the larger filter size could cause issues with clogging, next, we FAC sorted 2 test samples to remove any dead cells and debris. However, 10x sequencing then showed we lost all cardiomyocytes that we knew had gone into the FACS machine. As we had a small percentage of dead cells in the FAC sorted samples, we decided to perform the real experiment without FACS. The 10x machine did not get clogged running the samples and we obtained good cell numbers after sequencing (these numbers have now been added to the supplements as requested below). As explained above, we then used less stringent cut offs to be able to understand the data best, but we realise we should have redone this before adding the data to the manuscript, as this has caused the data to look less good. We have now redone the quality control and included all the data, which shows the quality of the data. Lines 752–758: Filter size justification and viability details added.

3. In the supplementary data, the number of cells and quality per sample should be stated to ensure the reader that quality was considered for each time point/sample-fish, and not just the final number of cells in the integrated dataset.

We have now added this information. Supplementary Fig. 6d–e shows cell counts per sample before and after QC filtering, and Supplementary Fig. 6a–b shows per-sample distributions of nCount_RNA, nFeature_RNA and mitochondrial percentage before and after filtering.

4. Considering, as mentioned at the beginning of this report, the lack of biological replicates. Also, in the supplementary materials, mention all the markers used for cell assignment, not just a few as in the plot Fig S1b, to ensure reproducibility.

We have now replaced Sup. Fig. 1b with a more extensive list and also added this for all sub plots. We acknowledge the limitation of single-fish time points and have addressed data quality as described in our general response above.

5. As mentioned before, authors should implement doublet filtering, and even consider tools like CellBender to improve the quality of the data.

We have now implemented doublet filtering using scDblFinder, which is well-validated for droplet-based scRNA-seq data and is well-suited to the tissue types in our dataset. Per-sample doublet classifications are shown in Supplementary Fig. 7c. We considered CellBender for ambient RNA removal but chose decontX, which we found to be well-calibrated for our data type and is computationally more tractable for our multi-sample integration

pipeline. Full details of all QC tools and parameters are provided in the updated Methods section.

Lines 113–114 / Figure 1b legend (Line 136): Total cell count updated from 85,516 to 60,598 cells, reflecting re-mapping to AstMex3 genome and updated QC pipeline. Lines 148–153 (Figure 1d legend): Explicit fold-change statistics added for leukocyte dynamics at each time point in both morphs.

Lines 762–764: scDblFinder and decontX explicitly stated

• Line 678: Missing mention of the number of individuals/replicates per treatment, for this and all the following experiments.

The number of individuals per group is now stated explicitly for all experiments throughout the Methods section and in the figure legends. This applies to all drug treatments, immunostaining experiments, and RNAscope analyses.

Line 678, all figure legends and Methods updated with n per group

• Line 781: Were different genome versions used for scRNA-seq and bulk RNA-seq, versions two and three, respectively? If so, both analyses should implement the same reference genome.

Yes, the bulk RNA-seq had been done with the new genome. We have now re-mapped the scRNAseq data to the same genome to improve comparisons.

Lines 919-925: Both scRNA-seq and bulk RNA-seq now mapped to AstMex3

Discussion:
The authors are missing key points or open questions and future directions; including those related to what the adaptive component of scar formation is in this particular cave and not in others of the few analysed.

We have expanded the Discussion to address open questions and future directions more explicitly. We now discuss the potential metabolic basis for the reduced adaptive immune capacity of Pachón and its potential link to the known metabolic remodelling in cave-adapted fish; the question of whether the impaired B cell response is specific to Pachón or shared across other cavefish populations, which remains an important open question; the molecular mechanisms by which B cells promote cardiomyocyte cell cycle progression, including candidate cytokine-mediated pathways; and the therapeutic implications of the two independent immune mechanisms identified. We acknowledge that only a small number of cavefish populations have been tested for heart regeneration capacity, making it premature to draw conclusions about whether the adaptive immune component of scar formation is a general feature of cave adaptation or specific to Pachón.

Lines 618–708: Metabolic basis, open questions, and therapeutic implications added

Line 1070: The presence of dead/dying cells as one of the clusters raises a huge concern, since you are still showing contamination in your data. In the methods, you stated removal of this ambient contamination, but it is still displayed on cell populations labelled

As described in our general response and the doublet comment above, in our new analysis these cells are not present anymore. We used less stringent cutoffs in our original analysis as the data is from injured samples, where you would expect a large number of dying cells in the samples just after injury and we wanted to capture any relevant data from this.

Summary of all the other changes made to the revised manuscript

Title page and author affiliations (Lines 1–14)

Affiliation 3 was updated to the new department of RR, LB, and RA. A new affiliation 4 was added for RA. Author order has been updated.

Abstract (Lines 31–46)

The abstract was substantially rewritten to more precisely describe the two immune mechanisms identified.

Lines 49–108 (Introduction): Multiple targeted edits as listed above; Lines 94–101 rewritten to include AMP and Acalabrutinib.
