# Supplementary material for: Absence of a prolonged macrophage and B cell response inhibits heart regeneration in the Mexican cavefish
Source: Nat Commun. 2026 Sep 17;17:10922. doi: 10.1038/s41467-026-77725-4 (PMC13680611; doi:10.1038/s41467-026-77725-4)
Supplement: Supplementary file 3 [file 41467_2026_77725_MOESM3_ESM.pdf]

nature portfolio

Corresponding author(s): M.T.M. Mommersteeg (mathilda.mommersteeg@dpag.ox.ac.uk)

Last updated by author(s): Jun 3, 2026

Reporting Summary

Nature Portfolio wishes to improve the reproducibility of the work that we publish. This form provides structure for consistency and transparency in reporting. For further information on Nature Portfolio policies, see our Editorial Policies and the Editorial Policy Checklist.

Statistics

For all statistical analyses, confirm that the following items are present in the figure legend, table legend, main text, or Methods section.

n/a	Confirmed	
☐	☒	The exact sample size (*n*) for each experimental group/condition, given as a discrete number and unit of measurement
☐	☒	A statement on whether measurements were taken from distinct samples or whether the same sample was measured repeatedly
☐	☒	The statistical test(s) used AND whether they are one- or two-sided<br>*Only common tests should be described solely by name; describe more complex techniques in the Methods section.*
☐	☒	A description of all covariates tested
☐	☒	A description of any assumptions or corrections, such as tests of normality and adjustment for multiple comparisons
☐	☒	A full description of the statistical parameters including central tendency (e.g. means) or other basic estimates (e.g. regression coefficient) AND variation (e.g. standard deviation) or associated estimates of uncertainty (e.g. confidence intervals)
☐	☒	For null hypothesis testing, the test statistic (e.g. *F*, *t*, *r*) with confidence intervals, effect sizes, degrees of freedom and *P* value noted<br>*Give P values as exact values whenever suitable.*
☒	☐	For Bayesian analysis, information on the choice of priors and Markov chain Monte Carlo settings
☒	☐	For hierarchical and complex designs, identification of the appropriate level for tests and full reporting of outcomes
☐	☒	Estimates of effect sizes (e.g. Cohen's *d*, Pearson's *r*), indicating how they were calculated

Our web collection on statistics for biologists contains articles on many of the points above.

Software and code

Policy information about availability of computer code

Data collection	Confocal images were acquired using Zeiss LSM 880 and Leica TCS SP8 AOBS systems. Brightfield histology was imaged on an Evident VS200 slide scanner. Sequencing was performed on the Illumina NextSeq 500/550. FASTQ files were generated using Cell Ranger mkfastq (v8.0.1).
Data analysis	scRNA-seq analysis was performed using Seurat v4.0.6, with batch correction via Harmony v1.0, ambient RNA removal via decontX, and doublet removal via scDblFinder. Pathway analysis used PROGENy v1.12.0 and fGSEA v1.16.0. Bulk RNA-seq was processed with STAR v2.7.11b and DESeq2. Deconvolution used BisqueRNA v1.0.5. Image quantification was performed in Fiji/ImageJ. Statistical analyses and visualisation used GraphPad Prism v10.1.1 and R v4.0.1. Full software versions are listed in the Key Resources table.

For manuscripts utilizing custom algorithms or software that are central to the research but not yet described in published literature, software must be made available to editors and reviewers. We strongly encourage code deposition in a community repository (e.g. GitHub). See the Nature Portfolio guidelines for submitting code & software for further information.

Data

Policy information about availability of data

All manuscripts must include a data availability statement. This statement should provide the following information, where applicable:

- Accession codes, unique identifiers, or web links for publicly available datasets
- A description of any restrictions on data availability
- For clinical datasets or third party data, please ensure that the statement adheres to our policy

Single-cell RNA sequencing data generated in this study are deposited in the NCBI Gene Expression Omnibus under accession number GSE291684. Bulk RNA-seq

data used for deconvolution analysis are available under GSE234990 (previously published; Lekkos et al., 2025). All data are publicly available with no restrictions. Data and material requests should be directed to M.T.M. Mommersteeg (mathilda.mommersteeg@dpag.ox.ac.uk). The Tg(Cau.ighv-ighm:EGFP) and rag1-hu1999 zebrafish lines are available from R. Richardson (rebecca.richardson@bristol.ac.uk).

Research involving human participants, their data, or biological material

Policy information about studies with human participants or human data. See also policy information about sex, gender (identity/presentation), and sexual orientation and race, ethnicity and racism.

Reporting on sex and gender	*Use the terms sex (biological attribute) and gender (shaped by social and cultural circumstances) carefully in order to avoid confusing both terms. Indicate if findings apply to only one sex or gender; describe whether sex and gender were considered in study design; whether sex and/or gender was determined based on self-reporting or assigned and methods used.* *Provide in the source data disaggregated sex and gender data, where this information has been collected, and if consent has been obtained for sharing of individual-level data; provide overall numbers in this Reporting Summary. Please state if this information has not been collected.* *Report sex- and gender-based analyses where performed, justify reasons for lack of sex- and gender-based analysis.*
Reporting on race, ethnicity, or other socially relevant groupings	*Please specify the socially constructed or socially relevant categorization variable(s) used in your manuscript and explain why they were used. Please note that such variables should not be used as proxies for other socially constructed/relevant variables (for example, race or ethnicity should not be used as a proxy for socioeconomic status).* *Provide clear definitions of the relevant terms used, how they were provided (by the participants/respondents, the researchers, or third parties), and the method(s) used to classify people into the different categories (e.g. self-report, census or administrative data, social media data, etc.)* *Please provide details about how you controlled for confounding variables in your analyses.*
Population characteristics	*Describe the covariate-relevant population characteristics of the human research participants (e.g. age, genotypic information, past and current diagnosis and treatment categories). If you filled out the behavioural & social sciences study design questions and have nothing to add here, write "See above."*
Recruitment	*Describe how participants were recruited. Outline any potential self-selection bias or other biases that may be present and how these are likely to impact results.*
Ethics oversight	*Identify the organization(s) that approved the study protocol.*

Note that full information on the approval of the study protocol must also be provided in the manuscript.

Field-specific reporting

Please select the one below that is the best fit for your research. If you are not sure, read the appropriate sections before making your selection.

☒ Life sciences ☐ Behavioural & social sciences ☐ Ecological, evolutionary & environmental sciences

For a reference copy of the document with all sections, see nature.com/documents/nr-reporting-summary-flat.pdf

Life sciences study design

All studies must disclose on these points even when the disclosure is negative.

Sample size	No formal power calculation was performed. Sample sizes were chosen based on previous experience with cardiac injury models in Astyanax mexicanus and zebrafish, and are consistent with published studies in the field. For histological quantifications, n=3–8 hearts per group per time point were used, which we consider sufficient to detect biologically meaningful differences given the effect sizes observed. For scRNA-seq, one pooled sample per condition was sequenced at high depth (~50,000 reads/cell) to maximise cell recovery and transcriptomic resolution.
Data exclusions	N/A
Replication	Key findings were validated using multiple independent approaches. The requirement for macrophage phagocytosis in regeneration was confirmed using two independent methods (Clodronate liposome depletion and pharmacological inhibition with 2-acetamidophenol). The role of B cells was validated in two species (surface fish and zebrafish) and using two independent experimental approaches (Acalabrutinib inhibition and rag1$^{-/-}$ genetic mutants). All experiments were performed on independent cohorts of animals.
Randomization	Following cryoinjury, fish were randomly assigned to treatment groups at the end of the surgical procedure. For comparisons between surface fish and Pachón cavefish, randomisation is not applicable as these are two naturally occurring morphs of the same species. All procedures were conducted at the same time of day to minimise technical variability.
Blinding	Investigators were blinded to group allocation during inhibitor treatments. All surgeries were performed without knowledge of treatment group. During analysis, a second investigator independently quantified a subset of images to validate the primary quantification, confirming consistent results.

Reporting for specific materials, systems and methods

We require information from authors about some types of materials, experimental systems and methods used in many studies. Here, indicate whether each material, system or method listed is relevant to your study. If you are not sure if a list item applies to your research, read the appropriate section before selecting a response.

Materials & experimental systems

n/a	Involved in the study
☐	☒ Antibodies
☒	☐ Eukaryotic cell lines
☒	☐ Palaeontology and archaeology
☐	☒ Animals and other organisms
☒	☐ Clinical data
☒	☐ Dual use research of concern
☒	☐ Plants

Methods

n/a	Involved in the study
☒	☐ ChIP-seq
☒	☐ Flow cytometry
☒	☐ MRI-based neuroimaging

Antibodies

Antibodies used	All primary and secondary antibodies are listed in the Key Resources table with supplier name and catalog number.
Validation	MF20 (DSHB AB_2147781) is a widely validated anti-myosin heavy chain antibody used extensively in zebrafish and teleost cardiac studies; cross-reactivity with A. mexicanus was confirmed by the expected pattern of cardiomyocyte labelling. Anti-PCNA (Dako M0879) and anti-Mef2 (Santa Cruz SC-313) are validated for use in zebrafish in prior published studies and showed expected nuclear staining patterns in both species. Anti-transgelin (Abcam ab14106) showed expected epicardial staining consistent with published expression patterns. Anti-GFP (Abcam ab13970) was used for detection of EGFP in the Tg(Cau.ighv-ighm:EGFP) zebrafish line, validated by the manufacturer and confirmed by colocalisation with EGFP fluorescence.

Animals and other research organisms

Policy information about studies involving animals; ARRIVE guidelines recommended for reporting animal research, and Sex and Gender in Research

Laboratory animals	Astyanax mexicanus surface fish (Río Choy background) and Pachón cavefish were used at 1–2 years of age. Zebrafish (Danio rerio, EKW/TL strain) were used at 1-2 years of age. Studies did not involve wild-caught animals.
Wild animals	Not applicable. All animals were laboratory-maintained.
Reporting on sex	Mixed-sex cohorts were used in all experiments to minimise potential sex-related bias. We acknowledge the inability to perform sex-based analyses as a limitation, and note that findings are expected to apply to both sexes based on the use of mixed cohorts.
Field-collected samples	Not applicable.
Ethics oversight	All A. mexicanus procedures were conducted under UK Home Office licences and approved by the University of Oxford's Committee on Animal Care and Ethical Review (ACER), in compliance with the UK Animals (Scientific Procedures) Act 1986 and EU Directive 2010/63/EU. Zebrafish experiments at the University of Bristol were conducted under UK Home Office licence and approved by the University of Bristol Animal Welfare and Ethical Review Body (AWERB).

Note that full information on the approval of the study protocol must also be provided in the manuscript.

Plants

Seed stocks	*Report on the source of all seed stocks or other plant material used. If applicable, state the seed stock centre and catalogue number. If plant specimens were collected from the field, describe the collection location, date and sampling procedures.*
Novel plant genotypes	*Describe the methods by which all novel plant genotypes were produced. This includes those generated by transgenic approaches, gene editing, chemical/radiation-based mutagenesis and hybridization. For transgenic lines, describe the transformation method, the number of independent lines analyzed and the generation upon which experiments were performed. For gene-edited lines, describe the editor used, the endogenous sequence targeted for editing, the targeting guide RNA sequence (if applicable) and how the editor was applied.*
Authentication	*Describe any authentication procedures for each seed stock used or novel genotype generated. Describe any experiments used to assess the effect of a mutation and, where applicable, how potential secondary effects (e.g. second site T-DNA insertions, mosiacism, off-target gene editing) were examined.*
